# Cerebellar aging is spatially heterogeneous and supports cognitive resilience in later life

**DOI:** 10.1038/s41593-026-02289-x

**Published:** 2026-06-10

**Authors:** Federico d’Oleire Uquillas, Esra Sefik, Jakob Seidlitz, Edan Daniel Hertz, Rafael Romero-Garcia, Varun Warrier, Richard A. I. Bethlehem, Aaron F. Alexander-Bloch, Jonathan D. Cohen, Samuel S.-H. Wang, Jorge Sepulcre, Patrizia Vannini, Jesse Gomez

**Affiliations:** 1 Princeton Neuroscience Institute, Princeton University, Princeton, NJ USA; 2 Lifespan Brain Institute, Children’s Hospital of Philadelphia, University of Pennsylvania, Philadelphia, PA USA; 3 Department of Psychiatry, University of Pennsylvania, Philadelphia, PA USA; 4 Department of Child and Adolescent Psychiatry and Behavioral Science, The Children’s Hospital of Philadelphia, Philadelphia, PA USA; 5 Department of Medical Physiology and Biophysics, Institute of Biomedicine of Seville (IBiS) HUVR/CSIC/University of Seville/CIBERSAM, ISCIII, Seville, Spain; 6 Department of Psychology, University of Cambridge, Cambridge, UK; 7 Department of Psychiatry, University of Cambridge, Cambridge, UK; 8 Gordon Center for Medical Imaging, Massachusetts General Hospital, Harvard Medical School, Boston, MA USA; 9 Martinos Center for Biomedical Imaging, Massachusetts General Hospital, Harvard Medical School, Boston, MA USA; 10 Department of Radiology and Biomedical Imaging, Yale School of Medicine, Yale University, New Haven, CT USA; 11 Brigham and Women’s Hospital, Harvard Medical School, Boston, MA USA

**Keywords:** Cognitive ageing, Cognitive neuroscience

## Abstract

The cerebellum contains most of the brain’s neurons and supports many functions, yet how it changes with age remains unclear. Here we used three brain imaging studies spanning 47,000 adults and examined how different parts of the cerebellum age and their relation to cognition. We characterized cerebellar aging using volumetry and the T1-weighted/T2-weighted ratio, and corroborated these findings with quantitative magnetic resonance imaging in an independent sample. We show a spatially heterogeneous pattern of aging in which specific association and motor-related regions show steeper relationships with age than other lobules. Greater cerebellar volume was associated with higher cognitive scores with increasing age, suggesting that cerebellar structure may provide brain reserve that helps maintain function despite aging. In patients with Alzheimer’s disease, cerebellar volume was linked to cognition in individuals with lower amyloid burden, especially in those carrying two copies of the *APOE*-ε4 risk gene. This supports a threshold-reserve model, in which the cerebellum helps sustain cognition until pathology becomes widespread. These results show that the cerebellum has an active role in healthy cognitive aging and resilience.

## Abstract (web summary)

The cerebellum ages unevenly, with some regions showing preservation that may help protect the brain from decline. The authors show that large cerebellar volume is associated with stronger cognitive resilience in healthy aging and Alzheimer’s disease.

## Main

Global population aging underscores the need to identify neural mechanisms supporting cognitive longevity. The number of individuals older than 70 years is increasing faster than that of younger adults^1^. Although research has focused largely on the cerebral cortex, converging evidence indicates that the cerebellum, long recognized for sensorimotor control, also supports higher-order cognitive and affective functions^2,3^. Through closed-loop circuits with cortical association areas and subcortical nuclei^4^, the cerebellum is positioned to influence cognition across the lifespan. Posterior cerebellar regions linked to prefrontal and parietal networks exhibit protracted maturation^5^, suggesting a potential role in late-life cognitive resilience.

The cerebellum is not spared by aging. Morphometric and postmortem studies show hemispheric and vermal shrinkage, and loss of Purkinje and granule cells^6,7^; longitudinal imaging shows accelerating atrophy in advanced age^8^. Aging effects appear particularly pronounced in posterior regions^9–11^, and cerebellar volume correlates with executive function and processing speed in older adults^10,11^. Yet, whether cerebellar variation contributes to cognitive reserve remains unclear because most prior studies used modest samples or coarse anatomical resolution, limiting assessment of spatial heterogeneity.

Reserve frameworks emphasize neural efficiency and adaptability as buffers against decline^12^, but focus largely on neocortical and hippocampal systems. The ‘dysmetria of thought’ hypothesis^13^ proposes that cerebellar dysfunction disrupts higher-order cognition analogously to motor dyscoordination, implying that degeneration may produce cognitive dysmetria whereas preserved cerebellar integrity may confer protection. The cerebellum has also been implicated in Alzheimer’s disease (AD)^14^, but while neuroimaging studies suggest cerebellar involvement in AD^15^, whether it contributes to pathology or resilience^16^ remains unclear. Some work even reports slower cerebellar than neocortical structural decay^17^, although those analyses treated the cerebellum as a single structure and may have overlooked regional heterogeneity. Reduced cerebellar volume across neurodegenerative disorders^18^ further underscores the need to clarify its role in aging.

In this study, we combine large-scale multimodal data from the Human Connectome Project (HCP) (HCP-Aging, *n* = 708), UK Biobank (UKB) (*n* = 45,013) and the Alzheimer’s Disease Neuroimaging Initiative (ADNI) (*n* = 1,423) to model cerebellar aging and its relationship to cognition, amyloid burden and the *APOE* genotype. We asked: Is cerebellar aging spatially heterogeneous? Do individual differences in cerebellar aging relate to cognitive resilience that is consistent with reserve? How do these processes manifest in AD dementia? By integrating normative and pathological aging, we position the cerebellum within broader frameworks of cognitive aging and reserve.

## Results

### Heterogeneous aging of cerebellar tissue and associations with cognition

**Fig. 1:**
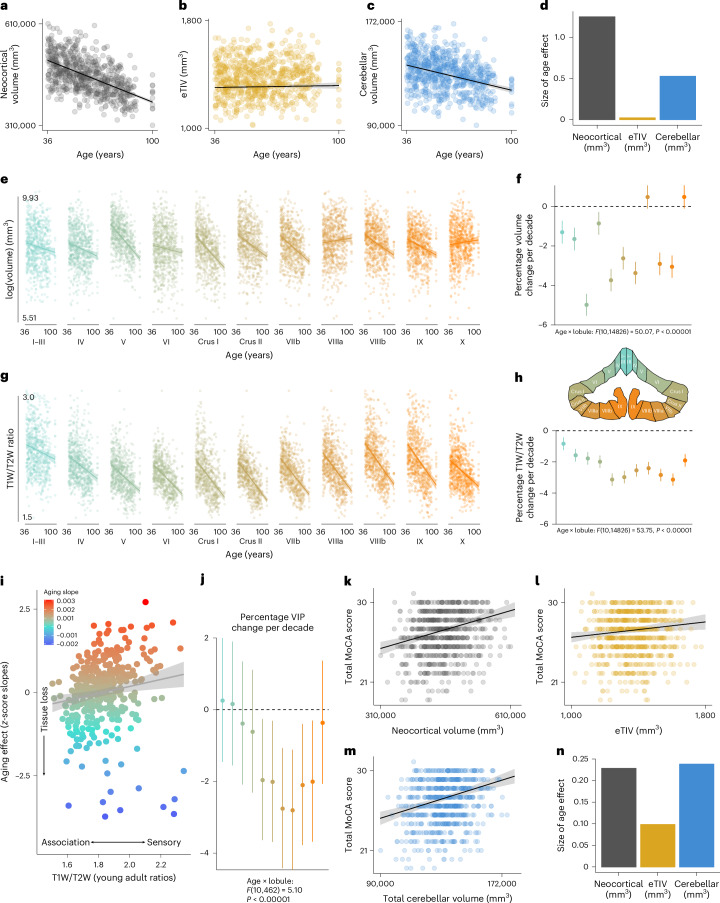
Cerebellar lobular heterogeneity and neocortical loss track cognitive differences with age. **a**,**c**, Linear regression models (two-sided *t*-tests of regression coefficients) correcting for biological sex, years of education and eTIV revealed significant inverse relationships between age and neocortical volume (*β* = −1,927.92 mm^3^ per year (−19,279 mm^3^ per decade), *P* = 0.00001, Cohen’s *f* = 1.27) (**a**), and cerebellar volume (*β* = −309.13 mm^3^ per year (−3,090 mm^3^ per decade), *P* < 2 × 10^−16^, Cohen’s *f* = 0.54) (**c**). **b**, Age and eTIV, correcting for sex and years of education, were not significantly related (*P* = 0.399). Solid lines represent ordinary least-squares regression fits (model-estimated mean response); shaded bands represent the 95% confidence intervals (CIs) of the regression estimate. All tests were two-sided. **d**, The magnitude of the age effects is summarized as Cohen’s *f* multivariate effects sizes. **e**, Aging-related effects on cerebellar lobular volume demonstrate heterogeneous age associations across lobules. The lines represent OLS regression fits (model-estimated mean volume), and the shaded bands denote the 95% CIs of the regression estimate. **f**, Lobule-specific age slopes are expressed as percentage volume change per decade derived from a linear mixed-effects model of log-transformed lobular volume. Analyses were performed in *n* = 707 independent participants, with the individual participant as the unit of analysis. The age × lobule interaction was evaluated using a two-sided Wald *F*-test from the mixed-model analysis of variance (ANOVA) (*F*(1,014,826) = 50.07, *P* < 2.2 × 10^−16^). The two-sided *t*-tests and 95% CIs used Satterthwaite-approximated degrees of freedom. *P* values for lobule-wise slopes and pairwise slope differences were adjusted for multiple comparisons using the Benjamini–Hochberg FDR. **g**, A similar pattern characterized the T1W/T2W signal, with strong inverse relationships along the rostro–caudal axis suggesting lower relative myelination in posterior parcels. Lines represent OLS regression fits (model-estimated mean signal); shaded bands represent the 95% CIs of the regression estimate. **h**, The inset cartoon of a coronal plane cerebellum shows the lobule colormap used in the scatterplot. Points show the estimated percentage change per decade derived from a linear mixed-effects model including an age × lobule interaction and the same covariates. Analyses were conducted in *n* = 707 independent participants. The age × lobule interaction was evaluated using a two-sided Wald *F*-test (*F*(1,014,826) = 53.75, *P* < 2.2 × 10^−16^). Lobule-specific slopes were estimated with two-sided *t*-tests and Satterthwaite-approximated degrees of freedom. Benjamini–Hochberg FDR correction was applied to multiple comparisons. Points represent model-estimated slopes. Whiskers denote the 95% CIs. **i**, Across 320 cortical ROIs, regions with lower young-adult T1W/T2W values (association-like cortex) showed more negative age-related slopes (greater tissue loss), whereas regions with higher T1W/T2W values (sensory-like cortex) showed flatter or positive slopes. Points represent ROI-specific regression slopes derived from two-sided general linear models. The line represents the model-estimated mean response; shaded bands denote the 95% CIs of the regression estimate. **j**, Lobule-specific age slopes in VIP are expressed as percentage volume change per decade, estimated from a linear mixed-effects model including an age × lobule interaction and controlling for sex and hemisphere. Analyses were conducted in *n* = 23 independent participants. The interaction was evaluated using a two-sided Wald *F*-test (*F*(10,462) = 5.10, *P* = 0.0000004). Lobule-specific slopes were estimated with two-sided *t*-tests and Satterthwaite-approximated degrees of freedom. Points represent model-estimated slopes. Whiskers denote the 95% CIs. **k**,**m**, Correcting for the same covariates as in **a**, greater MoCA total scores were associated with greater neocortical volume (*β* = 0.01 MoCA points per 1,000 mm^3^, *P* = 2.13 × 10^−9^, Cohen’s *f* = 0.23) (**k**), and greater cerebellar volume (*β* = 0.10 MoCA points per 1,000 mm^3^, *P* = 3.13 × 10^−10^, Cohen’s *f* = 0.24) (**m**). **l**, eTIV was also positively associated with cognition (*β* = 0.20 MoCA points per 100 cm^3^, *P* = 0.011, Cohen’s *f* = 0.10). Effects were estimated using linear regression models (two-sided *t*-tests of regression coefficients). Points represent model-estimated regression coefficients; shaded bands denote the 95% CI of the regression estimate. **n**, Differences in predictive strength across neocortical, eTIV and cerebellar volume are displayed as Cohen’s *f* effect sizes derived from the same regression models. Source data

In 708 neurotypical adults aged 36 to 100 years (Table 1), we examined age-related changes in cerebellar volume relative to the neocortex, controlling for biological sex, estimated total intracranial volume (eTIV) and years of education. Age was inversely associated to neocortical volume (*β* = −1,927.92, *P* = 0.00001, Cohen’s *f* = 1.27) and cerebellar volume (*β* = −309.13, *P* < 0.00001, Cohen’s *f* = 0.54), but not with eTIV (*P* = 0.399) (Fig. 1a–d).

To assess spatial heterogeneity, we parcellated the cerebellum into 11 rostrocaudal lobules. A mixed-effects model of log-transformed lobular volume (covariates: sex, eTIV, years of education, hemisphere) showed strong age-related volume associations and significant heterogeneity across lobules (age × lobule: *F*(1,014,826) = 50.07, *P* < 2.2 × 10^−16^) (Fig. 1e,f). Main effects were present for age (*F*(1,702) = 147.31, *P* < 2.2 × 10^−16^), lobule (*F*(1,014,826) = 65,756.26, *P* < 2.2 × 10^−16^), hemisphere (*F*(114,826) = 64.04, *P* = 1.31 × 10^−15^), sex (*F*(1,702) = 7.48, *P* = 0.006), eTIV (*F*(1,702) = 327.02, *P* < 2.2 × 10^−16^) and education (*F*(1,702) = 12.31, *P* = 0.0005). Pairwise slope comparisons were Benjamini–Hochberg-corrected (Supplementary Table 1).

Allowing hemisphere-specific slopes did not alter lobular heterogeneity (age x lobule: *F*(1,014,805) = 51.39, *P* < 2.2 × 10^−16^). There was no overall hemispheric difference in age slopes (age × hemisphere: *F*(114,805) = 1.53, *P* = 0.216) and no age × lobule × hemisphere interaction (*F*(1,014,805) = 1.27, *P* = 0.239) (Supplementary Fig. 1).

We next examined vermis regions using the same log-volume framework. Mixed-effects models revealed heterogeneous age slopes across vermis regions (age × vermis: *F*(42,820) = 4.56, *P* = 0.001), alongside a main effect of age (*F*(1,702) = 17.20, *P* = 0.00004) (Supplementary Fig. 2). eTIV (*F*(1,702) = 161.74, *P* < 2.2 × 10^−16^) and education (*F*(1,702) = 5.48, *P* = 0.019) were significant covariates, whereas sex was not (*P* = 0.166). Most vermis regions declined by approximately 0.9–1.5% per decade. Vermis VI, VII, IX and X showed significant negative estimates after Benjamini–Hochberg correction, whereas vermis VIII was near zero (Supplementary Table 2).

To assess whether volumetric reductions reflected loss of fine-scale tissue structure, we analyzed cerebellar T1-weighted (T1W)/T2-weighted (T2W) ratio maps, a contrast sensitive to myelin and neurite density^19,20^. Although T1W/T2W remains susceptible to cerebrospinal fluid partial-volume effects, it reduces intensity inhomogeneity and enhances tissue contrast. T1W/T2W declined with age across lobules (Fig. 1g): mixed-effects models (covariates: sex, eTIV, education and hemisphere) showed heterogeneous age slopes (age × lobule: *F*(1,014,826) = 53.75, *P* < 2.2 × 10^−16^), with main effects for lobule (*F*(1,014,826) = 2,819.43, *P* < 2.2 × 10^−16^), hemisphere (*F*(114,826) = 1,860.92, *P* < 2.2 × 10^−16^), and education (*F*(1,702) = 10.79, *P* = 0.001). Sex and eTIV were not significant (both *P* > 0.8). Percentage change per decade (Fig. 1h and Supplementary Table 3) showed significant decline across all regions after Benjamini–Hochberg correction, with the largest decreases in crus I (3.13%), crus II (2.98%) and lobule IX (3.14%), and the smallest in the anterior motor regions, consistent with a cerebellar sensory-association gradient^5^.

We next compared cerebellar aging to neocortical organization along the sensory-association hierarchy^21,22^. For each cortical region, we related mean T1W/T2W in 360 young adults (a proxy of sensory-association rank) to its age-related T1W/T2W slope in the aging cohort (Fig. 1i). Regions with lower baseline T1W/T2W—typically association cortices—showed more negative age slopes, whereas highly myelinated sensory regions were relatively preserved.

Because T1W/T2W is a relative metric, we validated these patterns using quantitative magnetic resonance imaging (qMRI) in an independent cohort (*n* = 23; 29–71 years). We derived maps of the volume of interacting protons (VIP), sensitive to tissue water macromolecular interactions expected to decline with tissue loss. Mixed-effects models controlling for sex and hemisphere showed significant lobular heterogeneity (age × lobule: *F*(10,462) = 5.10, *P* = 4.4 × 10^−7^) (Fig. 1j), with main effects of lobule (*F*(10,462) = 48.31, *P* < 2 × 10^−16^) and hemisphere (*F*(1,462) = 4.97, *P* = 0.026), but not age (*F*(1,20) = 3.61, *P* = 0.072). Simple slopes (Supplementary Table 4) showed reductions in crus I, crus II, VIIb, VIIIa, VIIIb and IX (uncorrected *P* < 0.05). After Benjamini–Hochberg correction, VIIb and VIIIa remained significant (*P* < 0.05), with crus I and II, VIIIb and IX trending (*P* < 0.1). A posterior (crus I and II) versus anterior (I–III, IV) contrast confirmed faster decline in posterior cognitive lobules (estimate = −0.002 per decade, *t* = −4.30, *P* < 0.0001), an approximately two percentage point greater decline per decade than the anterior regions. These qMRI findings replicate the posterior-biased pattern observed with T1W/T2W.

To clarify the biological interpretation of VIP, we examined age associations for tissue volume fraction (TV), water tissue fraction (WTF) and VIP across cerebellar lobules (Supplementary Fig. 9). TV and WTF exhibited complementary age-related patterns consistent with shifts in macromolecular versus water content across the adult lifespan. VIP demonstrated partially overlapping but distinct age associations and several lobules showed VIP age effects despite minimal TV or WTF slopes. These findings suggest that VIP captures microstructural variation beyond simple tissue loss or water accumulation, providing a complementary and partial-volume-robust index of cerebellar microstructural aging.

To assess whether cerebellar structure was related to cognition, older adults completed the Montreal Cognitive Assessment (MoCA), a battery sensitive to cognitive decline. Higher MoCA scores were associated with greater neocortical volume (*β* = 0.00001, *P* < 2.1 × 10^−9^, Cohen’s *f* = 0.23) (Fig. 1k) and greater cerebellar volume (*β* = 0.0001, *P* < 3.1 × 10^−10^, Cohen’s *f* = 0.24) (Fig. 1m). The eTIV measure was also positively associated with MoCA performance (*β* = 0.002, *P* = 0.011, Cohen’s *f* = 0.10) (Fig. 1l). Thus, greater global tissue volume, cortical and cerebellar, was related to cognitive performance in later life (Fig. 1n).

## Regional cerebellar age-related volume changes and associations with cognition

**Fig. 2:**
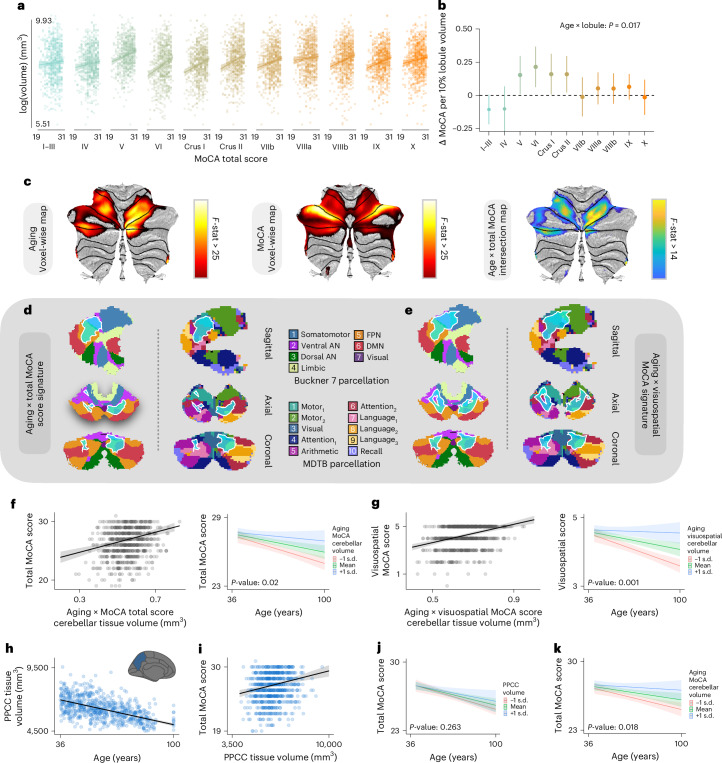
Cerebellar cognitive signature includes regions related to cerebello-cerebral networks and acts as a reserve factor. **a**, MoCA-related effects on ACAPULCO lobular volumes (mm^3^) demonstrate a heterogeneous pattern in the association between MoCA scores and cerebellar volume. Models were fitted to log-transformed lobular volume; lines represent model-estimated mean responses from linear mixed-effects models. Shaded bands denote the 95% CIs of the regression estimate. All tests were two-sided. **b**, The points represent lobule-specific regression coefficients derived from a multiple linear regression predicting MoCA total score from log-transformed lobular volumes, correcting for sex, eTIV, years of education and hemisphere. Analyses were conducted in *n* = 707 independent participants, with the individual participant as the unit of analysis. Points show model-estimated coefficients expressed as the change in MoCA score per 10% increase in lobular volume (model fit used unscaled mm^3^ predictors; coefficients scaled by log(1.10)). The error bars denote the 95% CIs of the parameter estimate derived from two-sided *t*-tests of the regression coefficients. An omnibus Wald test of equality of lobule coefficients was performed (two-sided *F*-test), assessing whether associations between lobular volume and MoCA differed across lobules (*F*(10,692) = 2.19, *P* = 0.0169). Pairwise differences between lobule coefficients were evaluated using Wald tests based on the model covariance matrix, with *P* values adjusted for multiple comparisons using the Benjamini–Hochberg FDR. Lobules IV, V, VI, crus I, crus II, VIIb, VIIIb, IX and X, showed positive associations, whereas I–III and VIIIa were positive but their CIs overlapped zero. **c**, VBM using modulated, normalized gray matter maps, provided *F*-test association maps of clusters in the cerebellar gray matter (the color intensity reflects the magnitude of the *F*-statistic). Top left: *F*-test association map of clusters in the cerebellar gray matter associated with years of age, correcting for eTIV (FWE-corrected *P* < 0.0001, qFDR = 0.002, *F*(1,705), *k* = 21). A brighter color (yellow) indicates greater years of age relating to lower cerebellar volume (mm^3^). Bottom left: *F*-test map of clusters in the cerebellar gray matter associated with MoCA total scores, correcting for eTIV (FWE-corrected *P* < 0.0001, qFDR = 0.002, *F*(1,705), *k* = 21). Right: conjunction map (FWE-corrected *P* < 0.0001, qFDR = 0.002, *F*(1,705), *k* = 21) visualizing the intersection of the aging and MoCA voxel-wise results, suggests that variations in this map contribute to aging-related changes in cognitive performance as measured by the MoCA. **d**,**e**, Resulting conjunction intersection clusters (FWE-corrected *P* < 0.0001, qFDR = 0.002, *F*(1,705), *k* = 21) of the aging-MoCA total score voxel-wise map (**d**) and the aging-MoCA-visuospatial subscale score map (**e**) overlaid on maps of Buckner 7 and Multimodal Domain Task Battery parcellations. The white outlines delineate the aging-MoCA mask in cyan. Overlaps illustrate qualitatively where clusters fall relative to broad functional zones. **f**,**g**, The lines represent model-estimated mean responses from the linear interaction models (two-sided tests of interaction terms); the shaded bands denote the 95% CIs of the regression estimate. **f**, Left: greater mean VBM-derived gray matter signal within the aging-total MoCA cerebellar signature mask was associated with higher total MoCA scores (*β* = 8.61 MoCA points per unit increase in signature value, *P* = 1.35 × 10^−10^, Cohen’s *f* = 0.25). **g**, Left: greater mean VBM-derived gray matter signal within the aging-visuospatial cerebellar signature mask was associated with higher MoCA-visuospatial scores (*β* = 3.86 score units per unit increase in signature value, *P* = 3.06 × 10^−16^, Cohen’s *f* = 0.32). **f**, Right: the negative association between age and MoCA scores was attenuated at higher levels of aging-MoCA signature (*P* = 0.02). **g**, Right: the aging-visuospatial cerebellar signature similarly moderated the association between age and visuospatial performance (*P* = 0.001). **h**–**k**, Post-hoc linear regression models, controlling for the same covariates, examined the relationship between PPCC volume, age, cognition and cerebellar reserve. **h**, Age was inversely related to PPCC volume (*β* = −30.25 mm^3^ per year, *P* < 2 × 10^−16^, Cohen’s *f* = 0.88). **i**, Higher PPCC volume was associated with greater total MoCA scores (*β* = 0.001 MoCA points per mm^3^, *P* = 3.71 × 10^−7^, Cohen’s *f* = 0.19). **j**, PPCC did not moderate the association between age and MoCA scores (*P* = 0.263). **k**, Aging-MoCA cerebellar signature remained a moderator of the age–MoCA association after including PPCC volume in the model (*P* = 0.018). Source data

Relating lobular gray matter volume (ACAPULCO regions of interest (ROIs)) to MoCA scores revealed heterogeneous structure cognition associations across lobules (Fig. 2a). In a joint log-volume model including all 11 bilateral lobules (adjusting for sex, eTIV, education and hemisphere), the MoCA × lobule interaction was significant (*F*(10,692) = 2.19, *P* = 0.0169), indicating spatially varying associations (Fig. 2b). Slopes are expressed as change in MoCA per 10% increase in lobular volume (ΔMoCA/10%), with Benjamini–Hochberg-adjusted *P* values. Lobules V and VI and crus I and II showed the strongest effects (VI: +0.27 (0.19, 0.35, 95% CI), *P* = 5.9 × 10^−10^; crus I: +0.27 (0.19, 0.35 CI), *P* = 3.0 × 10^−10^; crus II: +0.21 (0.14, 0.28 CI), *P* = 4.4 × 10^−8^) (Fig. 2b and Supplementary Table 5). An omnibus equality-of-slopes test confirmed heterogeneity (*F*(10,121.94) = 2.19, *P* = 0.017). A priori contrasts demonstrated stronger associations in posterior/association lobules (crus I, crus II) relative to anterior/motor lobules (I–III, IV) (difference in mean slope = +0.26 MoCA per 10% (95% CI 0.12, 0.41), *z* = 3.57, *P* = 0.0004). Thus, a 10% larger posterior lobule corresponded to roughly 0.2–0.3 higher MoCA points.

Voxel-based morphometry (VBM) corroborated these findings. Age-related gray matter associations were evident in the posterior cerebellum (family-wise error (FWE)-corrected *P* < 0.0001, false discovery rate-adjusted *q* value (qFDR) = 0.002, *F*(1,705), cluster extent *k* = 21 voxels) (Fig. 2c, top left); MoCA was associated with bilateral lobules V, VI, crus I and crus II (FWE-corrected *P* < 0.0001, qFDR = 0.002, *F*(1,705), *k* = 21) (Fig. 2c, bottom left). A conjunction analysis identified cerebellar cortex showing both aging and cognitive effects (Fig. 2c, right). Subscale analyses (Supplementary Fig. 3; unthresholded maps showing positive associations) revealed shared and distinct patterns. Only the visuospatial subscale survived multiple-comparisons correction (FWE-corrected *P* < 0.0001, qFDR = 0.002).

Network overlays showed conjunction clusters predominantly within association regions (frontoparietal, ventral-attention and portions of default mode) in the Buckner 7 Network parcellation^23^, and within attention and language representations as per Multimodal Domain Task Battery parcellation^2^, with some involvement of primary motor zones (Fig. 2d,e). Within these posterior regions, gray matter volume related to better cognition for both total MoCA (*β* = 8.61, *P* = 1.4 × 10^−10^, Cohen’s *f* = 0.25) and the visuospatial subscale (*β* = 3.86, *P* = 3.1 × 10^−16^, Cohen’s *f* = 0.32) (Fig. 2f,g).

MoCA scores declined linearly with age (*β* = −0.04, *P* = 9.7 × 10^−12^, Cohen’s *f* = 0.26). Therefore, we tested whether cerebellar volume moderated age–cognition associations. Cerebellar volume attenuated age–cognition associations (age × cerebellar volume: total MoCA *P* = 0.020; visuospatial MoCA *P* = 0.001; Fig. 2f,g). Individuals with cerebellar volume one standard deviation below the mean showed steeper negative age–cognition slopes, whereas those one standard deviation above the mean exhibited attenuated age–cognition slopes. This protective effect emerged after approximately 46 years of age for total MoCA and 44 years for visuospatial performance (simple slopes, positive FDR < 0.05). All moderation analyses were cross-sectional.

## Cerebellar versus cortical contributions to cognitive reserve

We next tested whether the reserve effect was specific to the cerebellum or shared by neocortical regions functionally connected to posterior cerebellar lobules VI, crus I and crus II. Based on established cerebello-cerebral networks, we defined a posterior midline cerebral cortex composite (precuneus-postcingulate cortex (PPCC)). Cerebellar and PPCC volumes were strongly correlated (*β* = 4,019.95, *P* = 2.0 × 10^−16^, Cohen’s *f* = 0.41), consistent with known subcortical-cortical networks^24^. While age was negatively associated with PPCC volume (*β* = −30.50, *P* = 2.0 × 10^−16^, Cohen’s *f* = 0.89) (Fig. 2h), and PPCC volume positively predicted MoCA (*β* = 0.001, *P* = 3.7 × 10^−7^, Cohen’s *f* = 0.19) (Fig. 2i), PPCC volume did not moderate cognition–age associations (PPCC × age: *P* = 0.263) (Fig. 2j). Importantly, when PPCC volume was included as a covariate in the cerebellar moderation model, the age × cerebellar volume interaction remained significant (*P* = 0.018) (Fig. 2k), indicating that cerebellar reserve effects are not solely explained by structurally coupled cortical regions.

We extended this analysis to additional network-matched cortical composites: a frontoparietal network (FPN) composite (rostral and caudal middle frontal, inferior parietal cortex) and a ventral-attention network (VAN) composite (insula, inferior frontal gyrus pars opercularis/triangularis, supramarginal cortex). Neither FPN nor VAN volume moderated age–MoCA associations (FPN × age: *P* = 0.188; VAN × age; *P*= 0.550). In models including both cerebellar and cortical interaction terms, the age × cerebellar volume effect remained significant or marginal (PPCC model: *β* = 0.014, *P* = 0.037; FPN model: *β* = 0.013, *P* = 0.056; VAN model: *β* = 0.016, *P* = 0.016), whereas cortical interaction terms remained nonsignificant (PPCC: *P* = 0.917; FPN: *P* = 0.972; VAN: *P* = 0.834).

We further tested canonical regions implicated in cognitive aging: the hippocampus and superior frontal cortex (Supplementary Fig. 4). Both showed expected negative age associations (hippocampus: *β* = −14.91, *P* < 2.0 × 10^−16^; superior frontal cortex: *β* = −87.82, *P* < 2.0 × 10^−16^), and positive associations with MoCA (hippocampus: *β* = 0.001, *P* = 0.00001; superior frontal cortex: *β* = 0.0002, *P* = 0.00001), yet neither moderated age–MoCA relationships (hippocampal volume × age: *P* = 0.906; superior frontal volume × age: *P* = 0.831). Critically, the age × cerebellar volume interaction remained significant after adjusting for hippocampal (*β* = 0.163, *P* = 0.021) or superior frontal volume (*β* = 0.169, *P* = 0.017). Together, these results demonstrate that cerebellar volume contributes unique protective variance to age-related cognition associations beyond structurally and functionally related neocortical regions.

### Assessment of sex effects on MoCA performance, education, eTIV and volume

In models correcting for eTIV and years of education, male and female HCP participants did not differ in neocortical volume (*P* = 0.990; male cortex mean volume: 478,998.40 mm^3^; female cortex mean volume: 438,824.30 mm^3^), cerebellar volume (*P* = 0.071; male cerebellar mean volume: 137,083.60 mm^3^; female cerebellar mean volume: 125,866.10 mm^3^), education (*P* = 0.501; male mean years of education: 17.80; female mean years of education: 17.23) or age (*P* = 0.980; male mean age: 60.35; female mean age: 59.82). As expected, females exhibited smaller eTIV (*β* = −73.83, Cohen’s *f* = 0.67, *P* = 2.0 × 10^−16^; male eTIV mean: 1,454.23 mm^3^; female eTIV mean: 1,302.61 mm^3^). Females also scored modestly higher on the MoCA (*β* = 0.494, Cohen’s *f* = 0.17, *P* = 0.00002; female MoCA average: 26.55; male MoCA average: 26.01).

We next tested whether sex moderated imaging–cognition associations. Sex did not interact with neocortical volume (*P* = 0.161), cerebellar volume (*P* = 0.912), age (*P* = 0.563), education (*P* = 0.093) or eTIV (*P* = 0.274) (Supplementary Fig. 5). Cerebellar VBM gray matter age maps, corrected for eTIV, were similar across sexes (Supplementary Fig. 5g, top; FWE-corrected *P* < 0.05). Although MoCA-related VBM maps showed a subtle right hemisphere bias, this effect did not survive correction for multiple comparisons (shown at uncorrected *P* < 0.0001; Supplementary Fig. 5g, bottom). Together, these findings indicate that the observed cerebellar aging and reserve effects are not driven by sex differences.

## Generalizability of cerebellar reserve to independent cognitive tasks

To test whether the aging-MoCA cerebellar signature generalized to independent measures, we examined four HCP tasks: dimensional card sorting task^25^; flanker attention task^26^; picture sequence memory (PSM) task^27^; and list sorting working memory (LSWM) task^28^. Interaction models controlling for sex, eTIV and education showed that greater cerebellar volume attenuated age-related decline in the card sorting (cerebellar volume × age: *β* = 0.99, *P* = 0.0005) and flanker task (cerebellar volume × age: *β* = 0.710, *P* = 0.005) (Supplementary Fig. 6a,b). Simple slopes (FDR < 0.05) indicated that protective effects emerged after approximately 55 (card sorting task) and 60 (flanker attention task) years (Supplementary Fig. 6b). Additive models showed positive main effects of cerebellar volume on both tasks (card sorting: *β* = 32.84, *P* = 1.1 × 10^−9^; flanker: *β* = 31.29, *P* = 3.5 × 10^−10^). By contrast, the aging-MoCA cerebellar signature volume did not moderate age-related performance for the PSM task (*P* = 0.646) (Supplementary Fig. 6c) or LSWM task (*P* = 0.441) (Supplementary Fig. 6d), although positive cerebellar volume main effects were observed in additive models (PSM model main effect of cerebellar volume: *β* = 45.81, *P* = 1.5 × 10^−7^; LSWM model main effect of cerebellar volume: *β* = 45.81, *P* = 1.5 × 10^−7^).

## Cerebellar cognitive reserve in the UKB

**Fig. 3:**
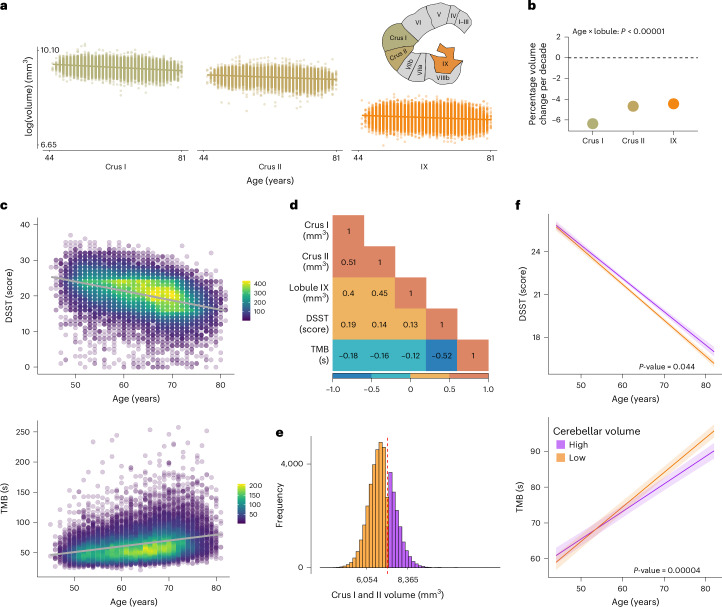
The cerebellum reproducibly modulates age-related cognitive differences in the UKB. Analyses were conducted in *n* = 35,763 independent participants with full complete data, with the individual participant as the unit of analysis. **a**, Replication of the HCP findings in the UKB cohort revealed heterogeneous age-related effects across crus I, crus II and lobule IX in linear models correcting for biological sex, total head volume and years of education. **b**, The points represent lobule-specific age slopes expressed as percentage volume change per decade, derived from a linear mixed-effects model of log-transformed lobular volume. The age × lobule interaction was evaluated using a two-sided Wald *F*-test from the mixed-model ANOVA (age × lobule: *F*(2,181,270) = 309.34, *P* < 2.2 × 10^−16^). Lobule-specific slopes were estimated with two-sided *t*-tests and the 95% CIs of the parameter estimate (smaller than the plotted data points). Estimated losses per decade were 6.36% for crus I (95% CI −6.36, −6.18), 4.68% for crus II (95% CI −4.87, −4.49) and 4.44% for lobule IX (95% CI −4.63, −4.25). **c**, Top: in linear regression models (two-sided *t*-tests), greater age was associated with lower DSST scores (*β* = −0.25 DSST points per year, *P* < 2 × 10^−16^, Cohen’s *f* = 0.41). **c**, Bottom: greater age was associated with longer completion time on the TMB (*β* = 0.93 seconds per year, *P* < 2 × 10^−16^, Cohen’s *f* = 0.31). The lines represent model-estimated mean responses from linear regression models; the shaded bands denote the 95% CIs of the regression estimate. **d**, Unadjusted Pearson correlations demonstrated associations among lobular volumes (crus I, crus II and lobule IX) and between cognitive measures. **e**, A Gaussian mixture model applied to a crus I–II composite identified two distinct distributions, enabling classification into lower-volume and higher-volume groups. **f**, Linear interaction models (two-sided tests of interaction terms) correcting for sex, years of education and total head volume revealed that individuals with higher cerebellar volume exhibited lower DSST scores (*P* = 0.044) and longer completion time on the TMB (*P* = 0.00004). The lines represent model-estimated mean outcomes from the interaction model; the shaded bands denote the 95% CIs of the regression estimate. Source data

We replicated our findings in an independent, large dataset of healthy older adults from the UKB (Table 2). Focusing on posterior lobules (crus I, crus II, lobule IX), we observed substantial age-related volume decline, most pronounced in crus I (*β* = −0.002, *P* < 2.0 × 10^−16^, Cohen’s *f* = 0.38), followed by crus II (*β* = −0.002, *P* < 2.0 × 10^−16^, Cohen’s *f* = 0.27) and lobule IX (*β* = −0.003, *P* < 2.0 × 10^−16^, Cohen’s *f* = 0.16) (Fig. 3a). In a joint mixed-effects model controlling for sex, hemisphere, education and total head volume, age effects differed across lobules (age × lobule: *F*(2,181,270) = 309.34, *P* < 2.2 × 10^−16^), with all main effects significant (all *P* < 3.5 × 10^−13^). Back-transformed estimates showed scaled-free decline across regions: crus I (−6.36% per decade, 95% CI −6.55, −6.18), crus II (−4.68%, 95% CI −4.87, −4.49) and lobule IX (−4.44%, 95% CI −4.63, −4.25) (Fig. 3b), replicating strong aging effects in the posterior cerebellum.

Cognitive performance declined with age for both digit symbol substitution task (DSST)^29^ (*β* = −0.25, *P* < 2.0 × 10^−16^, Cohen’s *f* = 0.41) and the trail-making test part B (TMB)^30^ (*β* = 0.93, *P* < 2.0 × 10^−16^, Cohen’s *f* = 0.31) (Fig. 3c). Posterior lobules showed the strongest task correlations (crus I: DSST *r* = 0.19, TMB *r* = −0.18; crus II: DSST *r* = 0.14, TMB *r* = −0.16; IX: DSST *r* = 0.13, TMB *r* = −0.12) (Fig. 3d).

Greater volume attenuated age-related decline for both the DSST (age × cerebellar volume: *β* = 0.01, *P* = 0.044) (Fig. 3e) and the TMB (age × cerebellar volume: *β* = −0.10, *P* = 0.00004) (Fig. 3f). That is, at age 80, individuals with higher cerebellar volume completed the TMB approximately 9 s faster and performed about 5% better on the DSST. Thus, in a large independent cohort, analysis of cerebellar structure replicated the heterogeneous aging across lobules and the protective moderation of age-related cognitive decline observed in the HCP, now in a mega-dataset of over 40,000 adults.

## Cerebellar reserve in AD

**Fig. 4:**
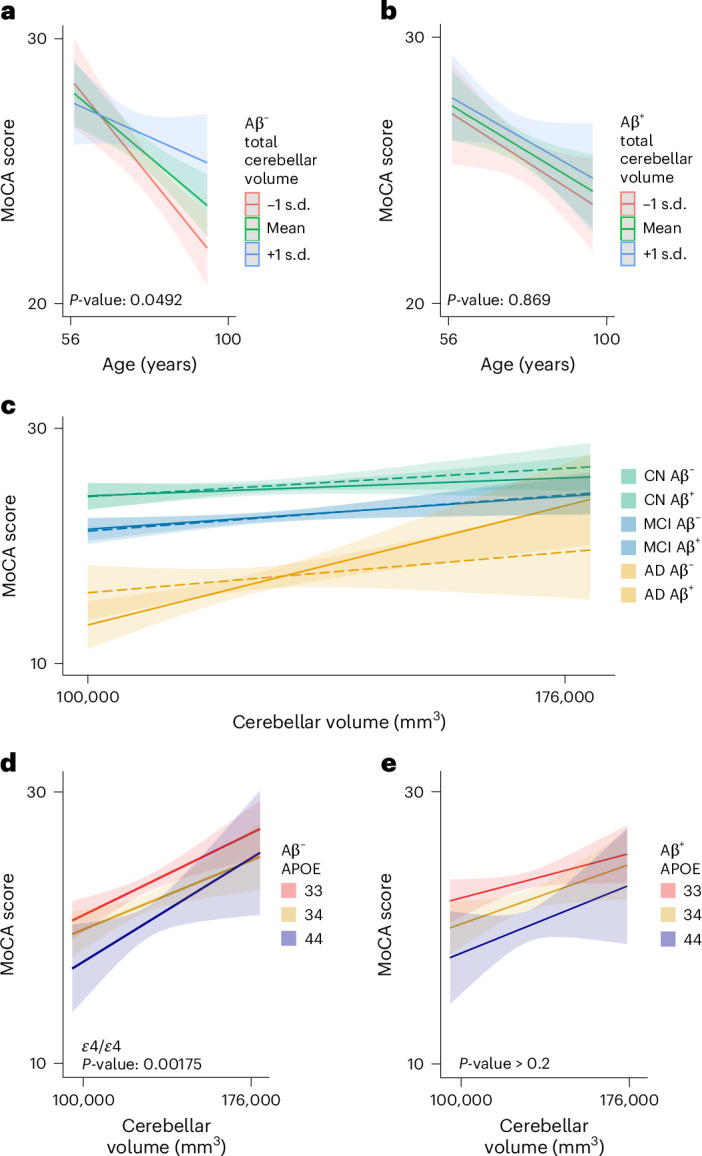
Cerebellar volume and MoCA score relationships in ADNI. Analyses were conducted in *n* = 1,350 independent participants from the ADNI cohort with complete data across all variables, with the individual participant as the unit of analysis. Linear models (two-sided tests of interaction terms) controlled for sex and eTIV. **a**,**b**, Predicted MoCA scores are shown as a function of age at three levels of total cerebellar volume (−1 s.d., mean, +1 s.d.), derived from linear interaction models (two-sided) including an age × cerebellar volume term. The lines represent model-estimated mean predicted MoCA scores; the shaded bands denote the 95% CIs of the regression estimate. **a**, In Aβ^−^ participants, the age × cerebellar volume interaction was significant (*P* = 0.049). **b**, In Aβ^+^ participants, the interaction was not significant (*P* = 0.869). Axes are scaled to the observed ranges. Lines and bands are model-based predictions. **c**, Stratified linear regression models examined cerebellar volume–MoCA associations according to amyloid and diagnostic status predicting MoCA scores. The lines represent model-estimated mean responses; the shaded bands denote the 95% CIs. In Aβ^−^ individuals (solid line), the association between cerebellar volume and MoCA was strongest among participants with an AD dementia clinical diagnosis (orange) (*P* = 0.004). The color indicates the diagnosis (CN: green, MCI: blue, AD: orange); line type indicates amyloid status (solid: Aβ^−^, dashed: Aβ^+^). **d**,**e**, Further stratification according to APOE genotype across diagnostic groups (*n* = 1,347 individual participants) revealed that among Aβ^−^ individuals (**d**), cerebellar volume was most strongly associated with MoCA scores in ε4/ε4 carriers (*P* = 0.00175). No significant *APOE* × volume effects were observed in Aβ^+^ individuals (**e**). Source data

We next tested whether cerebellar reserve extends to clinical populations using the ADNI (*n* = 1,423) (Table 3). Participants were stratified according to amyloid status (amyloid-negative, Aβ^−^; amyloid-positive, Aβ^+^) and cerebellar volume × age interactions were examined for MoCA scores, controlling for sex and eTIV. In Aβ^−^ individuals, larger cerebellar volume attenuated age-related MoCA slopes (cerebellar volume × age: *P* = 0.0492) (Fig. 4a). No moderation effect was detected in Aβ^+^ participants (*P* = 0.869) (Fig. 4b), consistent with a threshold-reserve model in which cerebellar structure buffers cognition when amyloid burden is low.

MoCA scores declined across diagnostic stage (cognitive normal (CN), mild cognitive impairment (MCI), AD; *F*(2) = 289.60, *P* < 0.0001), with all pairwise differences significant (mean MoCA score in CN: 25.07; MCI: 22.36; AD: 16.99) (Supplementary Fig. 7a). Neocortical volume similarly decreased according to stage (*F*(2) = 27.68, *P* < 0.0001; mean cortex volume in CN: 427,996.10 mm^3^; MCI: 423,554.40 mm^3^; AD: 395,070.80 mm^3^) (Supplementary Fig. 7b). In contrast, cerebellar volume did not differ across diagnosis (*F*(2) = 0.74, *P* = 0.475; mean cerebellar volume in CN: 127,668.20 mm^3^; MCI: 128,135.30 mm^3^; AD: 126,777.90 mm^3^) (Supplementary Fig. 7c).

We next examined cerebellar volume–cognition associations stratified according to diagnosis and amyloid status. In Aβ^−^ individuals, cerebellar volume interacted with diagnostic group in predicting MoCA (Aβ^−^ AD group: *β* = 0.0001, *P* = 0.004) (Fig. 4c and Supplementary Table 6). However, in a three-way model (age × cerebellar volume × group), no significant age moderation was observed within subgroups (all *P* > 0.3).

Given that *APOE* ε4 is a major AD risk factor, we tested whether cerebellar reserve persisted across genotype. Among Aβ^−^ individuals, greater cerebellar volume predicted higher MoCA scores across haplotypes, including ε4/ε4 homozygotes (Fig. 4d) (ε4/ε4: *β* = 0.001, *t* = 3.14, *P* = 0.00175; ε3/ε3: *β* = 0.002, *t* = 3.11, *P* = 0.00193; ε3/ε4: *β* = 0.001, *t* = 3.09, *P* = 0.00210). No significant effects were observed in Aβ^+^ participants (all *P* > 0.2) (Fig. 4e), indicating that cerebellar reserve operates primarily in the relative absence of substantial amyloid burden, including in genetically at-risk individuals.

Supplementary mixed-effects models stratified according to Aβ status (subject random intercepts, controlling for sex, eTIV and hemisphere), showed age × lobule interactions in both Aβ^−^ (*F*(1,014,763) = 15.35, *P* < 0.00001) and Aβ^+^ individuals (*F*(1,013,545) = 14.08, *P* < 0.00001). In both strata, posterior lobules (VI, VIIb, VIIIb, IX, crus I) showed steeper percentage-per-decade estimates than anterior lobules (I–IV), with the exception of strong effects in anterior lobule V (Supplementary Fig. 8a,b and Supplementary Table 7). Hemisphere did not moderate these effects (age × lobule × hemisphere: Aβ^−^ *P* = 0.220; Aβ^+^ *P* = 0.516).

Volume–MoCA associations were modest but spatially heterogeneous (volume × lobule: Aβ^−^ *F*(1,015,375) = 8.23, *P* < 0.00001; Aβ^+^ *F*(1,014,143) = 7.19, *P* < 0.00001) (Supplementary Table 8). In Aβ^−^ participants, positive ΔMoCA per 10% larger volume was observed in lobule IV (+0.24%, *P* = 0.003), V (+0.39%, *P* < 0.00001), VI (+0.21%, *P* = 0.008), crus I (+0.28%, *P* = 0.003), crus II (+0.15%, *P* = 0.029), VIIb (+0.45%, *P* < 0.00001) and IX (+0.16%, *P* = 0.003) (Benjamini–Hochberg-adjusted *P* < 0.05). In Aβ^+^ individuals, effects again concentrated in lobule V (+0.26%, *P* = 0.0008), crus I (+0.27%, *P* = 0.003) and VIIb (+0.42%, *P* < 0.00001), with most other regions null. Lobule X showed a small negative association (−0.25%, *P* = 0.001). Effects were modest (~0.2–0.45% MoCA per 10% larger volume).

Longitudinal ADNI analyses further showed that greater cerebellar volume predicted higher overall MoCA performance (*β* = 0.23, *P* = 0.04). Although volume × time was not significant in models with random intercepts and random slopes (*P* = 0.421), a significant interaction emerged in a random-intercept-only model (*β* = −0.031, *P* = 0.019), with higher initial cognitive performance and convergence over time. This pattern is consistent with a ‘front-loaded’ reserve model in which structural advantages buffer early cognitive differences.

## Discussion

Across neurotypical aging and clinical populations, we show that the cerebellum exhibits spatially heterogeneous senescence, with posterior association lobules and lobule V disproportionately vulnerable to tissue loss. This spatial heterogeneity was replicated in an independent, large-scale dataset, indicating that it is a robust feature of subcortical aging. Although the pattern echoes the sensory-association hierarchy of the neocortex, larger cerebellar volume attenuated age-related cognitive differences, consistent with a cerebellar contribution to brain reserve.

### Spatial heterogeneity of cerebellar aging

Prior morphometric studies suggested regional variability in cerebellar aging^9,10,31^ but lacked systematic lobular comparisons and microstructural resolution. Across large, multi-cohort datasets and complementary tissue metrics, we identified a reproducible posterior-skewed pattern of aging, with association lobules (notably crus I and crus II) exhibiting steeper age-related effects than anterior motor lobules, with the notable exception of lobule V (Fig. 1e). This spatial pattern broadly mirrors functional cerebellar organization and provides a high-resolution, cross-cohort characterization of cerebellar aging.

The steep volumetric decline in lobule V, an anterior sensorimotor region, contrasted with stronger posterior microstructural effects in crus I and crus II, suggesting that volumetric and tissue-composition metrics capture partly distinct biological processes. Lobule V volume loss, which was observed in both the HCP and ADNI datasets (Supplementary Fig. 8), may reflect macroscopic degeneration, whereas posterior microstructural changes may reflect alterations in myelination or synaptic density. Intrinsic physiology may also contribute: Purkinje cells in the anterior lobules (III and IV) exhibit greater excitability than those in nodular regions such as lobule X^32^; within lobule V, zebrin-positive and zebrin-negative modules differ markedly in firing rates, with zebrin-negative cells firing roughly twice as fast^33^. Anterior lobules are also particularly susceptible to alcohol-related degeneration in adults^34^, suggesting that lobule V may lie at a physiological intersection where elevated excitability and environmental vulnerability increase cumulative metabolic burden across the lifespan.

Microstructural analyses further indicated that age-related cerebellar volume loss reflects tissue-content changes rather than uniform shrinkage. Cerebellar lobules showed reductions in tissue indices with age, whereas neocortical sensory regions exhibited higher T1W/T2W values, plausibly reflecting iron accumulation^35^. Cortical association areas instead displayed trajectories resembling posterior cerebellar lobules, consistent with coordinated vulnerability across distributed association systems. Preferential decline of later-maturing cerebellar regions is also consistent with retrogenesis^36,37^, whereby developmentally protracted systems are disproportionately susceptible to degeneration. Together, these complementary findings indicate that cerebellar aging is spatially organized, developmentally patterned and biologically grounded.

### Cerebellar structure and cognitive performance

Cerebellar aging was behaviorally meaningful. Across cohorts, higher cognitive performance, including MoCA, processing speed and executive measures, was associated with greater cerebellar volume, particularly in posterior lobules representing frontoparietal, default-mode and ventral-attention networks^10^ (Fig. 2d). Cerebellar volume also moderated age–cognition associations, such that individuals with larger cerebella exhibited attenuated age-related cognitive differences (Fig. 2f,g). This effect persisted after controlling for canonical cortical markers of aging, including hippocampal, superior frontal and association-cortical volumes, and was not observed for these cortical comparators. The same moderation pattern was replicated in UKB executive measures (Fig. 3f), reinforcing that cerebellar integrity supports maintenance of cognitive function across independent samples. Together, these convergent findings align with cerebellar scaffolding frameworks^38^, in which cerebellar circuits stabilize distributed cortical computations as neocortical systems decline, positioning the cerebellum as a structural substrate of cognitive resilience in later life.

### Cerebellar contributions along the AD continuum

Our findings align with accumulating evidence implicating the cerebellum in MCI and AD dementia. Although historically considered relatively spared, the cerebellum exhibits amyloid, tau pathology and microgliosis^39^, and structural or functional alterations even in the prodromal stages^14,15,40^. Posterior cerebellar regions aligned with association networks appear particularly vulnerable^15^, paralleling network-selective degeneration in the cerebrum; cerebellar structure has been linked to proxies of cognitive reserve in MCI^41^.

Stratification according to amyloid status revealed that cerebellar volume was related to cognitive resilience primarily in individuals with a lower amyloid burden (Fig. 4a), which is consistent with a threshold-reserve framework^42^. Cerebellar atrophy was evident across the amyloid spectrum, yet the strongest cognitive associations emerged in patients with clinically diagnosed AD with a lower amyloid burden (Fig. 4c) and in *APOE* ε4/ε4 carriers (Fig. 4d), suggesting that cerebellar structural integrity may buffer cognitive symptoms early in the disease trajectory before compensatory capacity is exceeded.

Consistent with this interpretation, cerebellar structure remained relatively stable across diagnostic clinical stages (Supplementary Fig. 7), contrasting with pronounced neocortical atrophy. Moderation effects were most evident in Aβ^−^ individuals, in line with evidence that reserve mechanisms operate preferentially at the earlier stages of pathology^43^. Together, these findings suggest that cerebellar structural integrity may contribute to cognitive resilience across the continuum from normative aging to early AD.

### Genetic and mechanistic considerations

The regional vulnerability observed in this study likely reflects convergent effects of glial physiology, network connectivity and genetic risk. Beyond *APOE* ε4, AD-associated loci, including *CLU*, *CR1* and *PICALM*, implicate lipid metabolism, endocytosis and microglia-mediated immune pathways^44^. The cerebellum, and posterior lobules, densely interconnected with association cortex, are metabolically demanding and enriched in oligodendrocyte^45^ and microglial populations^46,47^ that may heighten susceptibility. Notably, cerebellar microglia exhibit a transcriptionally distinct, immune-enriched and metabolism-enriched profile and show pronounced age-related shifts relative to other brain regions^46^.

Iron accumulation, a recognized driver of degeneration^48,49^, may further amplify oxidative stress, microglial activation and myelin disruption. *APOE* ε4 could exacerbate these processes through impaired lipid homeostasis and heightened neuroinflammatory signaling^50^. Such mechanisms provide a plausible basis for posterior lobular vulnerability and amplified effects in genetic risk carriers.

### Longitudinal and methodological context

Although our analyses were cross-sectional, the cerebellar volume–cognition association was replicated across three independent cohorts. Exploratory ADNI analyses further suggested that individuals with larger cerebella maintained better cognitive performance across visits, consistent with a reserve effect. Several limitations warrant consideration. The cross-sectional design limited inference about the temporal relationships between cerebellar atrophy and cognitive decline, and MoCA’s global scope limited domain-specific interpretation. Aging effects were spatially complex rather than strictly anterior-posterior, and incomplete education and socioeconomic data limited formal modeling of reserve proxies. Further, future multimodal longitudinal studies integrating structural, functional and biomarker measures will be essential to clarify how cerebellar structure supports cognitive resilience over time.

### Implications and conclusions

Across three large cohorts, cerebellar aging showed a reproducible pattern with strongest effects in the posterior lobules and lobule V. Cerebellar volume predicted cognitive resilience in normative aging and AD samples, positioning the cerebellum as an active component of brain aging. Extending reserve frameworks, cerebellar integrity appeared to buffer cognition, particularly before substantial amyloid burden, which is consistent with scaffolding and threshold-reserve models in which structural resources sustain function until pathology overwhelms compensation. Therefore, cerebellar structure may mask emerging symptoms despite advancing cortical pathology, potentially delaying detection under cortex-centric models. Integrating cerebellar metrics into neurodegeneration models, and targeting cerebellar-cortical circuits therapeutically, may strengthen strategies to preserve cognitive health.

## Methods

### Participants and cohorts

In this cross-sectional study, we sourced MRI data from three cohorts (Table 1) spanning the adult lifespan: the HCP-aging (*n* = 708; aged 36–100), the UKB (*n* = 45,013; aged 44–81) and the ADNI (*n* = 1,423; aged 56–95), totaling 47,144 participants. Sex distributions are reported in Tables 1–3 (HCP-aging, female = 400 (56.5%); UKB, 52.9% female; ADNI sex counts are provided for each diagnostic and amyloid cell in Table 3). All participants provided informed consent and were compensated. Studies were conducted under institutional review board approval.

**Table 1:** Sample demographics and imaging characteristics in the HCP-aging sample

	HCP-aging sample
*n*	708
Age (years)	60.05 (15.59)
Sex, *n* (% female)	400 (56.5)
Total cerebellar volume (mm^3^)	130,746.01 (13,494.55)
Total neocortical volume (mm^3^)	456,301.18 (51,798.86)
eTIV (mm^3^)	1,520,809.62 (188,357.16)
MoCA total score	26.32 (2.52)
MoCA-visuospatial score	4.14 (0.98)
Years of education	17.48 (2.21)
Adjusted household income	63,681.88 (62,350.68)

Data are reported as the mean (s.d.) unless stated otherwise.

**Table 2:** Sample demographics and imaging characteristics in the UKB sample

	UKB sample
*n*	45,577
Age (years)	63.60 (7.55)
Sex, *n* (% female)	19,474 (52.9)
Cerebellar volume (mm^3^)	6,963.05 (874.29)
Total brain volume (mm^3^)	1,054,072.94 (128,909.05)
Years of education, *n* (%)	
Professional	20,552 (49.7)
NVQ, HND, HNC	5,760 (13.9)
CSEs	8,607 (20.8)
O Levels	1,854 (4.5)
A Levels	2,415 (5.8)
College	2,177 (5.3)
DSST correct matches score	20.24 (4.96)
TMB (s)	64.19 (23.31)
Mean household income, *n* (%)	
Less than 18,000	2,935 (9.9)
Less than 31,000	858 (2.9)
Less than 52,000	4,707 (15.9)
Less than 100,000	8,921 (30.1)
Greater than 100,000	12,261 (41.3)

Data are reported as mean (s.d.) unless stated otherwise. Sex (count/%) is reported per cohort; ADNI is presented according to diagnostic/amyloid subgroup. A Level, Advanced Level (13 years of study); CSE, Certificate of Secondary Education; HNC, Higher National Certificate; HND, Higher National Diploma; NVQ, National Vocational Qualification; O Level, Ordinary Level (11 years of study).

**Table 3:** Sample demographics and imaging characteristics in the ADNI sample

	CN AB^−^	CN AB^+^	MCI AB^−^	MCI AB^+^	AD AB^−^	AD AB^+^	*P* value
*n*	303	280	140	123	80	62	
Age (years)	74.50 (6.52)	75.09 (6.66)	77.01 (8.08)	75.59 (7.38)	75.59 (8.15)	75.60 (7.71)	0.028
Sex, *n* (% female)	168 (55.4)	157 (56.1)	56 (40.0)	45 (36.6)	30 (37.5)	28 (45.2)	<0.001
Total cerebellar volume (mm^3^)	128,536.33 (12,534.48)	126,742.07 (11,900.30)	125,587.07 (12,507.90)	127,262.08 (13,347.47)	126,333.72 (11,792.44)	127,340.50 (11955.39)	0.277
Total neocortical volume (mm^3^)	429,978.70 (45,107.06)	425,880.94 (43,210.13)	403,448.18 (50,591.46)	410,926.35 (56,019.73)	389,682.71 (48,569.70)	401,895.65 (48,685.18)	<0.001
eTIV (mm^3^)	1,451,505.59 (156,887.52)	1,438,760.03 (149,726.34)	1,465,296.78 (157,303.30)	1,482,354.46 (158,795.24)	1,422,662.74 (175,772.87)	1,474,327.26 (178,113.72)	0.060
Years of education	16.32 (2.78)	16.40 (2.65)	15.45 (2.89)	15.56 (3.44)	15.27 (2.66)	15.62 (2.75)	0.086
MoCA total score	24.94 (2.15)	25.21 (1.83)	20.21 (5.20)	20.52 (6.08)	16.87 (4.39)	17.13 (5.02)	<0.001
CDR							<0.001
0	263 (87.1)	241 (86.4)	18 (12.9)	11 (8.9)	0 (0)	1 (1.6)	
0.5	37 (12.3)	38 (13.6)	85 (60.7)	82 (66.7)	22 (27.5)	19 (30.6)	
1	2 (0.7)	0 (0)	28 (20.0)	20 (16.3)	52 (65.0)	30 (48.4)	
2	0 (0)	0 (0)	8 (5.7)	8 (6.5)	6 (7.5)	11 (17.7)	
3	0 (0)	0 (0)	1 (0.7)	2 (1.6)	0 (0)	1 (1.6)	

Data are reported as the mean (s.d.) unless stated otherwise.

### Demographics

We included participants with complete cerebellar MRI coverage and cognitive testing. In HCP-aging, there were two (0.28%) American Indian or Alaskan Native individuals, 52 (7.20%) Asian, 98 (13.84%) Black or African American, 506 (71.61%) White, 36 (4.38%) with more than one ethnicity and 14 (1.98%) individuals with unknown or unreported ethnicity, with 69 (9.75%) identifying as Hispanic or Latino, 632 (89.27%) not Hispanic nor Latino and seven (0.28%) of unreported ethnic group. We also sourced MRI data from the UKB but did not have access to ethnicity or ethnic group status. Additionally, the ADNI cohort consisted of two (0.14%) American Indian or Alaskan Native individuals, seven (0.50%) Asian, 22 (1.55%) Black or African American, one (0.07%) Native Hawaiian or other Pacific Islander, 670 (47.08%) White, 11 (0.77%) individuals of more than one ethnicity and 710 (49.89%) individuals of unknown or unreported ethnic status, with 16 (1.12%) identifying as Hispanic or Latino, 694 (48.77%) not Hispanic or Latino and 713 (50.11%) of unreported ethnic group.

### Clinical characterization (ADNI)

For ADNI participants, a clinical diagnosis of MCI or AD was ascertained by a consensus of trained clinicians, considering cognitive scores including MoCA^51^, the Clinical Dementia Rating Scale^52^ (score of 0 for CN function, 0.5 for MCI, 1 for mild dementia, 2 for moderate dementia and 3 for severe and advanced dementia) and the Alzheimer’s Disease Inventory. Our ADNI analysis considered a total of 565 Clinical Dementia Rating (CDR) = 0, 672 CDR = 0.5, 147 CDR = 1, 33 CDR = 2 and four CDR = 3 individuals. The ADNI sample was stratified according to a CN, MCI and AD dementia clinical diagnosis. We opted to include individuals with a ‘subjective cognitive concerns’ in the CN group, given previous reports showing that optimal subgroup classification of ADNI data using spectral embedding, multidimensional scaling, uniform manifold approximation and projection and *t*-distributed stochastic neighbor embedding, is best captured by the CN, MCI and AD diagnostic groupings^53^.

### Cognitive assessments

Cognitive assessments were tailored based on the cohort; they included the MoCA^51^ within 365 days of the MRI procedures, the DSST^29^ and TMB^30^, tests of processing speed and attention, and the Clinical Dementia Rating Scale^52^. No MoCA cutoff was imposed for inclusion. Among the HCP-aging participants included in the analyses, MoCA ranged from 19 to 30 (Q1 = 25, median = 27, mean = 26.32, Q3 = 28). This wide variability is consistent with a community sample without a MoCA-based inclusion threshold.

MoCA scores below 26 in the HCP-aging cohort reflect expected variability in community samples, as optimal screening cutoffs differ across cohorts (for example, Nasreddine et al.^51^). Nonetheless, lower scores may include individuals with an early or prodromal pathology. Similarly, Aβ^−^ yet cognitively impaired ADNI participants may represent non-AD conditions (for example, suspected non-AD pathology), with heterogenous trajectories^54^. Although amyloid-stratified analyses mitigate this concern, we interpret cerebellar–cognition associations conservatively and do not infer causality.

### MRI and positron emission tomography acquisition

All participants underwent 3T T1-weighted MRI. Structural images were segmented using FreeSurfer (v.7.1.1) and ACAPULCO^55^ for cerebellar parcellation in HCP and ADNI, and FMRIB software library-derived morphometry for the UKB (see Miller et al.^56^ and Alfaro-Almagro et al.^57^ for processing details). The cortex was parcellated using the HCP-MMP atlas^19^.

In ADNI, amyloid-PET [^61^C]-Pittsburgh compound B was acquired 50–70 min after injection and processed centrally to derive partial-volume-corrected regional uptake value ratios. The *APOE* genotype was determined from blood samples using standard assays. Additional acquisition parameters are provided in Supplementary Table 9.

### Statistical analysis

We quantified associations between sample characteristics using R (v.4.0.4). Multivariate linear regression models were conducted across the HCP, UKB and ADNI cohorts, covarying for biological sex, eTIV (from FreeSurfer) or total brain volume (from the FMRIB software library), and years of education for the HCP and the UKB samples. Robust standard error estimates were calculated unless otherwise specified; standardized *β*-coefficients are reported for all models along with Cohen’s *f* effect sizes for partial effects in multivariate regression.

### Lobule-wise aging models (HCP)

In the HCP cohort, we investigated relationships between age, total cerebellar volume (ACAPULCO parcellation), total neocortical volume and eTIV from FreeSurfer, and MoCA scores. Lobular gray matter volumes (mm^3^) were log-transformed and modeled using mixed-effects models with age × lobule interactions, adjusting for sex, eTIV and years of education.

Primary analyses used bilateral summed homologous lobules, defined a priori to (1) increase signal to noise, (2) reduce multiple comparisons and (3) align with our focus on lobule-level aging gradients rather than hemispheric lateralization.

Type III ANOVA tables were computed using Satterthwaite approximations for the numerator and denominator degrees of freedom and are reported as *F*(numerator d.f., denoninator d.f.). All tests were two-sided. Lobule-specific age slopes were estimated on the log scale and converted to percentage change per decade as 100 × (exp(10*β*^) − 1). CIs used asymptotic (*z*) inference. Left–right slope differences were tested on the log scale and corrected using Benjamini–Hochberg FDR (*q* < 0.05). Hemisphere-specific age × lobule × hemisphere models (Supplementary Fig. 1) were performed analogously.

### Vermis analyses (HCP)

Supplementary analyses examined the mid-sagittal vermis regions (ACAPULCO: Vermis VI–X). Log-transformed volumes were modeled using linear mixed-effects models with age × vermis ROI interactions, adjusting for sex, eTIV and years of education.

A Type III ANOVA with Satterthwaite degrees of freedom tested the age × vermis ROI interaction. Vermis-specific age slopes were estimated and converted to percentage change per decade as 100 × (exp(10*β*^) − 1). We report point estimates and 95% asymptotic (*z*-based) CIs and provide Benjamini–Hochberg-adjusted pairwise slope comparisons. Percentage-per-decade estimates are visualized in Supplementary Fig. 2 and reported in Supplementary Table 2.

### Microstructure analyses: T1W/T2W (HCP)

To index cerebellar tissue microstructure, we analyzed voxel-wise T1W/T2W ratio maps, a unitless contrast sensitive to myelin, neurite content and partial-volume effects content^19,20^. In the cerebellum, this measure is influenced by tissue microstructural content and intra-voxel cerebrospinal fluid admixture. Ratios were computed within ACAPULCO-defined lobules. We hypothesized reduced age effects in sensorimotor lobules and stronger decline in posterior association lobules.

Log-transformed T1W/T2W values were modeled using linear mixed-effects models with age × lobule interactions, adjusting for sex, eTIV, years of education and hemisphere, with subject random intercepts. Type III tests with Satterthwaite degrees of freedom provided omnibus inference. Lobule-specific age slopes were estimated and expressed as percentage change per decade: 100 × (exp(10*β*^) − 1). Pairwise slope differences were Benjamini–Hochberg FDR-corrected.

To compare cerebellar and cortical tissue aging patterns, we analyzed 320 cortical ROIs from the HCP Multimodal Parcellation (HCP-MMP1^19^) using HCP-aging T1W/T2W maps. For each ROI, age-related slopes were estimated with linear models, and mean T1W/T2W from the HCP young-adult reference group (aged 22–35 years) indexed baseline tissue properties. A general linear model was used to test whether baseline T1W/T2W predicted age-related decline across ROIs. The fitted regression and 95% CIs are shown in Fig. 1i.

### qMRI validation cohort

To validate the T1W/T2W findings, qMRI data were acquired from 23 healthy adults (aged 29–71) at the Princeton Neuroscience Institute (3T Siemens Prisma scanner, 64-channel head coil). Multi-flip angle fast low-angle shot three-dimensional scans (FLASH) were acquired with a repetition time of 19 ms, echo time of 3.34 ms and flip angles 4, 10 and 20. Echo-planar inversion recovery scans had a repetition time of 2,920 ms and inversion times of 2,400, 1,200, 400 and 200 ms. For each participant, FLASH and EPI images were processed using a MATLAB pipeline (mrQ^58^), including gradient nonlinearity correction, bias field correction and co-registration to individual T1 images. We analyzed the VIP, a quantitative index of water protons interfacing with cellular structures.

Proton-density-derived VIP maps were normalized to Montreal Neurological Institute space, parcellated with ACAPULCO, and averaged within lobules (normalization visually verified). Age was mean-centered (years). Sex was included as a covariate. Because VIP reflects voxel-wise tissue properties rather than regional volume, intracranial volume was not modeled. Log-transformed VIP values were analyzed using linear mixed-effects models with age × lobule interactions and subject random intercepts. Type III *F*-tests with Satterthwaite degrees of freedom provided omnibus inference. Lobule-specific age slopes were estimated and expressed as percentage change per decade: 100 × (exp(10*β*^) − 1), with Benjamini–Hochberg FDR correction across lobules. A planned contrast compared posterior cognitive lobules (crus I and II) against motor lobules (I–III, IV), using balanced weights on age slopes (averaged over the hemisphere). Model diagnostics showed approximately normal residuals and no singular fits.

qMRI measures were derived to characterize cerebellar microstructure beyond volume. These included TV, indexing macromolecular tissue content, WTF (WTF = 1 – TV) and VIP, a composite metric of tissue microstructure after accounting for free water. Because TV and WTF explicitly model tissue and water contributions, they are less susceptible to cerebrospinal fluid-related partial-volume effects, particularly in thin folia. VIP builds on this framework and is expected to be more robust than heuristic ratios such as T1W/T2W. For supplementary analyses, lobular qMRI measures were averaged across hemispheres, and age associations were tested using linear models correcting for sex.

### Lobule-wise cognition models (HCP)

To assess the relationship between cognition and cerebellar volume at the lobular level, bilaterally summed ACAPULCO volumes were log-transformed and entered into multiple regression models predicting MoCA. Covariates included sex, eTIV and years of education. Coefficients were rescaled to reflect ΔMoCA per 10% increase in volume. 95% CIs were derived from the model standard errors.

Pairwise slope differences were tested using Wald contrasts from the model’s variance–covariance matrix and corrected using Benjamini–Hochberg FDR. Guided by cerebellar functional organization, we contrasted posterior association lobules (crus I and crus II) with anterior and motor lobules (I–III, IV, V) using equal-weight linear combinations, reported as ΔMoCA per 10% volume with CIs.

### VBM analyses (HCP)

VBM was performed in CAT12^59^ (https://neuro-jena.github.io/cat/). CAT12 uses a standardized pipeline optimized for VBM analyses in SPM12^60^, including bias correction of field intensity non-uniformities and tissue segmentation with partial-volume modeling. Analyses used modulated, normalized maps (nonlinear modulation only), with eTIV as a covariate. Statistical conjunction of corrected maps was performed in FreeSurfer to identify regions associated with age and cognition (FWE-corrected *P* < 0.05).

In supplementary analyses, voxel-wise associations between cerebellar gray matter and individual MoCA subscales were tested in CAT12/SPM, adjusting for eTIV (Supplementary Fig. 3). Maps are displayed as (positive) signed values on the SUIT flatmap^61^, with green contours marking *P* < 0.001 (uncorrected) (Supplementary Fig. 3). The group-level voxel-wise SPM T-map shows reduced coverage in inferior cerebellar regions. This reflects conservative group-level masking during CAT12/SPM second-level estimation (for example, implicit intersection masking across individuals and tissue probability thresholds), rather than insufficient cerebellar coverage in the underlying data. Visual inspection of individual-level scans and cerebellar parcellations confirmed appropriate inclusion and segmentation of inferior cerebellar lobules across participants.

### Cortical comparators and reserve modeling (HCP)

Cortical comparators included bilateral Desikan–Killiany composites for the PPCC/DMN (precuneus and posterior cingulate), FPN (rostral middle frontal, caudal middle frontal, inferior parietal) and VAN (insula, pars opercularis, pars triangularis, supramarginal). Gray matter volumes (mm^3^) were *z*-scored within sample. Age was mean-centered. Covariates included sex, eTIV and years of education.

Reserve was tested via age × volume interactions predicting MoCA. Single-moderator models included one ROI at a time. Dual-moderator models included both the cerebellar MoCA signature and a cortical ROI with corresponding interaction terms. We report interaction coefficients, *t* statistics and 95% CIs. Moderation effects were compared directly using Wald tests, with Benjamini–Hochberg correction across related tests.

To test combinatorial reserve effects, we fitted linear models including age × cerebellum × cortex (cortex = PPCC/DMN, FPN or VAN composites), with all lower-order terms and covariates (sex, eTIV, years of education). The three-way interaction term indexed age-slope modulation according to regional volume. Robustness was evaluated using HC3 heteroskedasticity-consistent standard errors and Benjamini–Hochberg correction across cortical ROIs.

Specificity analyses examined bilateral hippocampal and superior frontal volumes (Desikan–Killiany). We tested age–volume associations, volume–MoCA associations, age–volume interactions on MoCA and whether cerebellar volume × age effects persisted after controlling for hippocampal or superior frontal volumes (Supplementary Fig. 4). All models included sex, eTIV and years of education as covariates. Interaction effects were visualized using simple slopes at −1 s.d., mean and +1 s.d. of the moderator.

### Sex interaction analyses (HCP)

We also tested sex interactions with key predictors using prespecified linear models. MoCA ~ age × sex + eTIV + years of education; MoCA ~ years of education × sex + eTIV; MoCA ~ neocortical volume × sex + eTIV + years of education; MoCA ~ eTIV × sex + years of education; MoCA ~ cerebellar volume × sex + eTIV + years of education (Supplementary Fig. 5). For the voxel-wise cerebellar VBM gray matter analyses, we repeated age-related and MoCA-related models stratified according to sex (with eTIV as covariate); the age effect VBM map survived correction for multiple comparisons (Supplementary Fig. 5g, top) (FWE-corrected *P* < 0.05), while the MoCA effect VBM map did not and is shown in Supplementary Fig. 5g (bottom) as uncorrected at *P* < 0.0001.

### Task-level validation (HCP)

In HCP-aging, we tested whether the aging-MoCA cerebellar ROI moderates age-related differences in NIH Toolbox performance: dimensional card sorting task^25^, flanker inhibitory control & attention task^26^, PSM^29^ and LSWM^28,62^. For each task, we fitted ordinary least-squares linear models: score ~ age × cerebellar volume + sex + eTIV + years of education, with primary inference on the age × cerebellar volume interaction (Supplementary Fig. 6). Interaction effect sizes are reported as Cohen’s *f*. Significant interactions were probed using simple-slope/Johnson–Neyman procedures (two-sided and FDR-adjusted within the task). To assess additive structure–performance associations, we also fitted models without the interaction term.

### UKB validation analyses

In the UKB validation sample, we examined associations between cerebellar volume and age, and cognitive performance (DSST^29^; TMB^30^ tests). Lobule-specific aging effects were tested for crus I, crus II and lobule IX (left/right hemispheres retained) using mixed-effects models. Lobular volumes were log-transformed to estimate percentage change per decade: 100 × (exp(10*β*^) − 1).

Type III tests with Satterthwaite degrees of freedom provided omnibus inference. Lobule-specific age slopes were estimated and expressed as percentage per decade with 95% CIs; pairwise slope differences were examined with Benjamini–Hochberg correction for multiple comparisons. Gaussian mixture models^20^ classified participants into high-volume and low-volume subgroups based on mean crus I and crus II volume.

### Age, cerebellar volume and cognition across amyloid and *APOE* (ADNI)

In ADNI, we examined associations among cerebellar and neocortical volumes, age, *APOE* genotype and MoCA scores in CN participants, and participants with MCI and AD dementia. Analyses were first stratified according to amyloid status (Aβ^−^/Aβ+), testing the moderation of the cross-sectional age–MoCA association according to total cerebellar volume: MoCA ~ age × cerebellar volume + sex + eTIV. Education or socioeconomic status were not included because of high missingness or unavailability. Predicted lines were generated at −1 s.d., mean and +1 s.d. of cerebellar volume (95% CIs; two-sided).

We then fitted three hypothesis-driven models using diagnostic group according to amyloid status (CN Aβ^−^, CN Aβ^+^, MCI Aβ^−^, MCI Aβ^+^, AD Aβ^−^, AD Aβ+): (1) MoCA ~ cerebellar volume × group; (2) MoCA ~ age × cerebellar volume × group; (3) MoCA ~ cerebellar volume × *APOE* zygosity. All adjusting for age, sex and eTIV. Given the limited number of a priori models, *P* values are reported unadjusted. Sample sizes were: 303 CN Aβ^−^; 280 CN Aβ^+^; 355 MCI Aβ^−^; 336 MCI Aβ^+^; 80 AD Aβ^−^; and 62 AD Aβ^+^. In the Aβ^−^ group, there were 352 *APOE* 3/3 carriers, 252 3/4 carriers and 54 4/4 carriers. In the Aβ^+^ group, there were 331 3/3 carriers, 214 3/4 carriers and 65 4/4 carriers.

### Lobule-wise age and cognition effects (ADNI)

Within the Aβ^−^ and Aβ^+^ strata, we fitted linear mixed-effects models with individual random intercepts to test hemispheric lobule-specific aging effects: log(volume) ~ age × lobule × hemisphere + sex + eTIV. Age slopes were estimated and expressed as percentage change per decade 100 × (exp(10*β*^) − 1), with 95% CIs and Benjamini–Hochberg-adjusted pairwise comparisons.

To examine lobule-specific volume–cognition associations, we modeled MoCA as a function of log-transformed lobule volume (separately within Aβ^−^ and Aβ^+^), adjusting for covariates (Supplementary Fig. 8). Effects were expressed as ΔMoCA per 10% greater lobule volume, with 95% CIs and Benjamini–Hochberg correction.

### Longitudinal cognition models (ADNI)

To test whether baseline cerebellar volume predicts longitudinal cognitive change, we fitted linear mixed-effects models using all available MoCA assessments across visits for every individual. Models included time since baseline (years; mean-centered per participant), baseline cerebellar volume, age, sex and eTIV, with participant as a random intercept. Random slope models additionally included participant-specific slopes for time: MoCA = time × cerebellar volume + age + sex + eTiV + (1|individual) + (0 + time|individual). We also evaluated a simpler random-intercept-only model. Baseline MoCA was not included as a covariate given evidence that repeated cognitive testing can lead to practice-related score improvements that do not reflect true cognitive change^63,64^. Therefore, including baseline MoCA can obscure the genuine longitudinal relationship between brain structure and cognitive trajectory by absorbing interindividual variance that partly reflects learning effects. Finally, we note that individuals who remain in longitudinal studies longer often represent a healthier survivor subgroup^65,66^, which can attenuate brain–behavior associations over time.

### Reporting summary

Further information on research design is available in the Nature Portfolio Reporting Summary linked to this article.

## Online content

Any methods, additional references, Nature Portfolio reporting summaries, source data, extended data, supplementary information, acknowledgements, peer review information; details of author contributions and competing interests; and statements of data and code availability are available at 10.1038/s41593-026-02289-x.

## Supplementary information

Supplementary Information Supplementary Methods, Figs. 1–9 and Tables 1–9.

Reporting Summary

## Source data

Source Data Statistical source data for Figs. 1–4.

## Data Availability

Data analyzed are available at public repositories, which may require appropriate data sharing agreements from each individual publicly accessible database, including LONI for ADNI and HCP data (https://ida.loni.usc.edu/), and biobank UK for UKB data (https://biobank.ctsu.ox.ac.uk). Deidentified data for Figs. 1–4 can be found at https://github.com/fdoleireuquillas/AgingCerebellum. Source data are provided with this paper.

## Code availability

Source code related to the statistical analysis and visualization can be found at https://github.com/fdoleireuquillas/AgingCerebellum.
