# Author Correction: Cerebellar aging is spatially heterogeneous and supports cognitive resilience in later life

**DOI:** 10.1038/s41593-026-02374-1

**Published:** 2026-06-23

**Authors:** Federico d’Oleire Uquillas, Esra Sefik, Jakob Seidlitz, Edan Daniel Hertz, Rafael Romero-Garcia, Varun Warrier, Richard A. I. Bethlehem, Aaron F. Alexander-Bloch, Jonathan D. Cohen, Samuel S.-H. Wang, Jorge Sepulcre, Patrizia Vannini, Jesse Gomez

**Affiliations:** 1 Princeton Neuroscience Institute, Princeton University, Princeton, NJ USA; 2 Lifespan Brain Institute, Children’s Hospital of Philadelphia, University of Pennsylvania, Philadelphia, PA USA; 3 Department of Psychiatry, University of Pennsylvania, Philadelphia, PA USA; 4 Department of Child and Adolescent Psychiatry and Behavioral Science, The Children’s Hospital of Philadelphia, Philadelphia, PA USA; 5 Department of Medical Physiology and Biophysics, Institute of Biomedicine of Seville (IBiS) HUVR/CSIC/University of Seville/CIBERSAM, ISCIII, Seville, Spain; 6 Department of Psychology, University of Cambridge, Cambridge, UK; 7 Department of Psychiatry, University of Cambridge, Cambridge, UK; 8 Gordon Center for Medical Imaging, Massachusetts General Hospital, Harvard Medical School, Boston, MA USA; 9 Martinos Center for Biomedical Imaging, Massachusetts General Hospital, Harvard Medical School, Boston, MA USA; 10 Department of Radiology and Biomedical Imaging, Yale School of Medicine, Yale University, New Haven, CT USA; 11 Brigham and Women’s Hospital, Harvard Medical School, Boston, MA USA

**Keywords:** Cognitive ageing, Cognitive neuroscience

Correction to: *Nature Neuroscience* 10.1038/s41593-026-02289-x, published online 10 June 2026.

In the version of the article initially published, in Fig. 4d, the *P*-value was incorrect and the data was duplicated from that in Fig. 4e. Fig. 4d has now been corrected in the HTML and PDF versions of the article, as seen in Fig. 1.

**Fig. 1:**
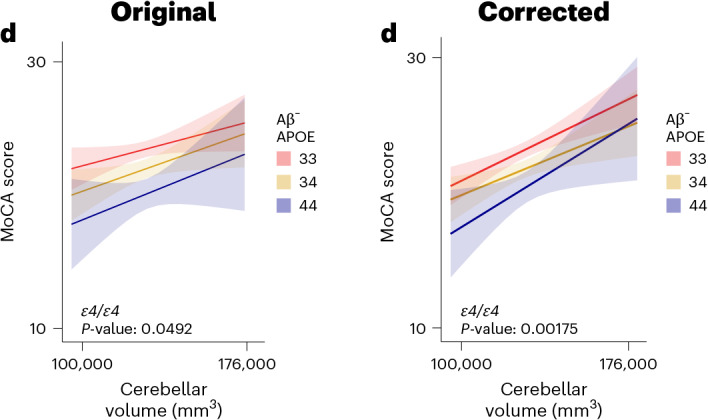
**Original and corrected Fig. 4d.**

