# Supplementary material for: Cerebellar aging is spatially heterogeneous and supports cognitive resilience in later life
Source: Nat Neurosci. 2026 Jun 10;29(7):1699–710. doi: 10.1038/s41593-026-02289-x (PMC13337503; doi:10.1038/s41593-026-02289-x)
Supplement: Supplementary file 1 — Supplementary Methods, Figs. 1–9 and Tables 1–9. [file 41593_2026_2289_MOESM1_ESM.pdf]

nature neuroscience

Supplementary information https://doi.org/10.1038/s41593-026-02289-x

Cerebellar aging is spatially heterogeneous and supports cognitive resilience in later life

In the format provided by the authors and unedited

Supplementary Material for

Cerebellar aging is spatially heterogenous and supports cognitive resilience in later life

d'Oleire Uquillas et al., 2026 Nature Neuroscience

Table of Contents

Supplementary Methods	…	pgs.	2
Supplementary Figures	…	pgs.	3 – 11
Supplementary Tables	…	pgs.	12 – 18

Supplementary Methods

Montreal Cognitive Assessment

The MoCA is a quick screening instrument for mild cognitive impairment, ranging from 1-30 points, and administered in 10-15 minutes, during which different cognitive domains are measured: Short-term memory recall, delayed memory recall, visuospatial abilities (clock drawing, 3D cube copy), processing speed, working memory (sustained attention task, serial subtraction task, and digits forward and backward memory task), language (3-item confrontation naming task, repetition of 2 syntactically complex sentences, and a verbal fluency task), and orientation (to time and place where being evaluated).

ADNI Funding

Data collection and sharing was funded by the Alzheimer's Disease Neuroimaging Initiative (ADNI) (National Institutes of Health Grant U01 AG024904) and DOD ADNI (Department of Defense award number W81XWH-12-2-0012). ADNI is funded by the National Institutes of Aging, the National Institute of Biomedical Imaging and Bioengineering, and through generous contributions from the following: AbbVie, Alzheimer's Association, Alzheimer's Drug Discovery Foundation, Araclon, Biotech, BioClinica, Biogen, Bristol-Myers Squibb, CereSpir, Cogstate, Eisai, Elan Pharmaceuticals, Eli Lilly, EuroImmun, F. Hoffman-La Roche and its affiliated company Genentech, Fujirebio, GE Healthcare, IXICO, Janssen Alzheimer Immunotherapy Research & Development, Johnson & Johnson Pharmaceutical Research & Development, Lumosity, Lundbeck, Merck, Meso Scale Diagnostics, NeuroRx Research, Neurotrack Technologies, Novartis Pharmaceuticals, Pfizer, Piramal Imaging, Servier, Takeda Pharmaceutical, and Transition Therapeutics. The Canadian Institutes of Health Research provide funds to support ADNI clinical sites in Canada. Private section contributions are facilitated by the Foundation for the National Institutes of Health (www.fnih.org). The grantee organization is the Northern California Institute for Research and Education, and the study is coordinated by the Alzheimer's Therapeutic Research Institute at the University of Southern California. ADNI data are disseminated by the Laboratory for Neuro Imaging at the University of Southern California.

Supplementary Figures

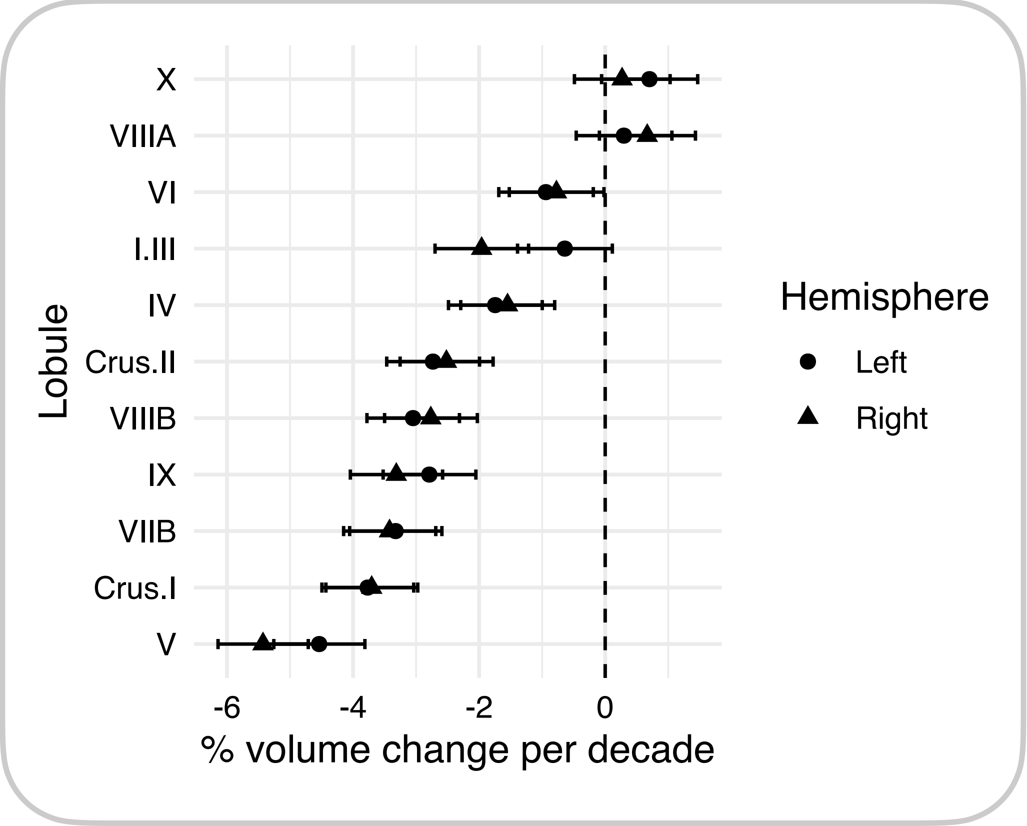

Supplementary Figure S1. Hemisphere-specific cerebellar lobule age slopes. Points denote the model-estimate percentage change in lobular volume per decade for the left (filled circles) and right (filled triangles) cerebellar hemispheres derived from a linear mixed-effects model including Age × ROI × Hemisphere interaction terms, correcting for biological sex, years of education, and estimated total intracranial volume (eTIV). Analyses were conducted in n=707 independent human participants, with the individual participant serving as the unit of analysis. Horizontal caps represent 95% confidence intervals (CIs) of the parameter estimate, and the short connector links the left and right estimates within each lobule. Most lobules show reliable age-related decline, indicated by CIs below zero. There was no global difference in age slopes between hemispheres (Age × Hemisphere: $F(1,14805)=1.53$, $p=0.216$) and no evidence that hemisphere modulates lobule-specific age slopes (Age × Lobule × Hemisphere: $F(10,14805)=1.27$, $p=0.239$). All tests were two-sided.

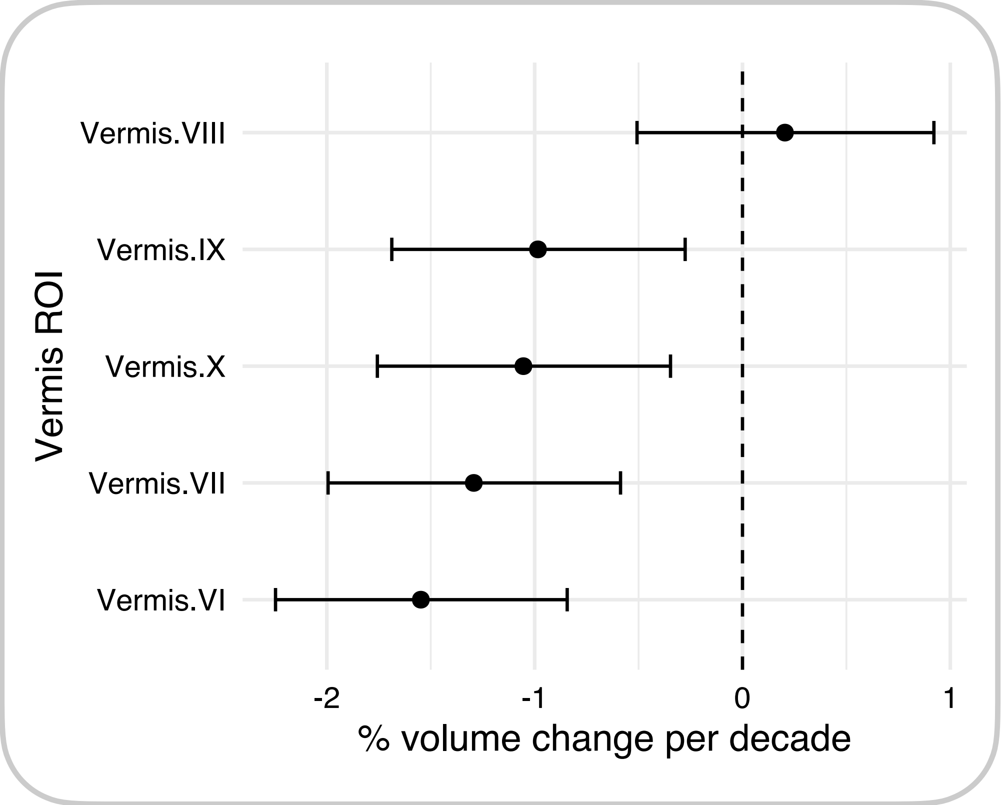

Supplementary Figure S2. Age-related vermis decline. Points denote the model-estimated percentage change in lobular volume per decade for vermis lobules VI-X derived from a linear mixed-effects model including Age × ROI interaction terms, correcting for biological sex, estimated intracranial volume (eTIV), and years of education. Analyses were conducted in n=707 independent participants, with the individual participant serving as the unit of analysis. Error bars represent 95% confidence intervals (CIs) of the parameter estimate, and the dashed vertical line indicates 0% change per decade. The mixed-effects model revealed a significant Age × Vermis interaction, indicating heterogeneous age-related slopes across vermis regions ($F(4,2820)=4.56$, $p=0.001$), along with a main effect of age ($F(1,702)=17.20$, $p=0.00004$). Expressed on a relative scale, most vermis regions showed decline of approximately 0.9–1.5% per decade. Vermis VI, VII, IX and X exhibited negative estimates with 95% CIs below zero after Benjamini-Hochberg correction, whereas Vermis VIII showed an estimate near zero with a CI spanning zero. Benjamini-Hochberg-adjusted pairwise comparisons and exact percent change per decade for each ROI are provided in **Table S2**. All tests were two-sided.

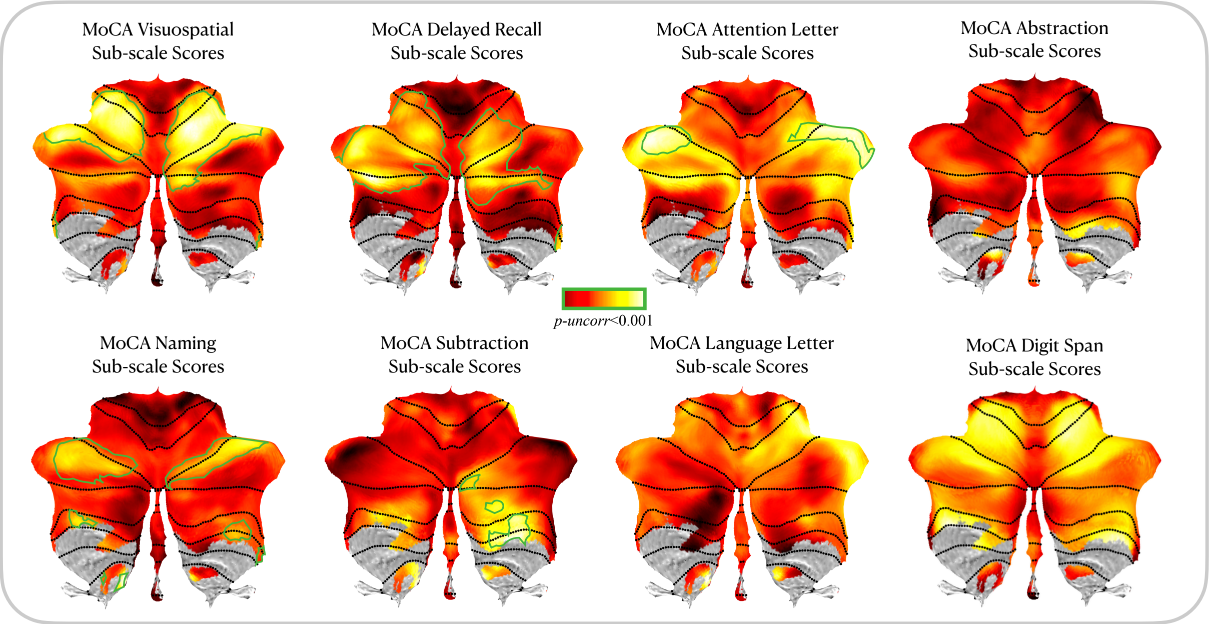

Supplementary Figure S3. Unthresholded cerebellar voxel-based morphometry maps for MoCA sub-scales. Each panel shows the signed contrast estimate from voxel-wise linear regressions relating MoCA sub-scale scores on cerebellar gray matter volume derived from CAT12 segmentation, with estimated intracranial volume (eTIV) included as a covariate. Analyses were conducted in n=708 independent participants, with the individual participant serving as the unit of analysis. Statistical maps are displayed on the SUIT cerebellar flatmap, where warmer colors indicate higher cognitive scores associated with greater gray matter volume. Only positive contrasts are shown. The Visuospatial sub-scale yielded clusters surviving whole-cerebellum multiple-comparisons correction (voxel-wise FWE $p<0.0001$; qFDR=0.002). All other panels are shown unthresholded, with green contours indicating voxels reaching *p-uncorrected*<0.001 for visualization. Black dotted curves denote cerebellar lobular boundaries.

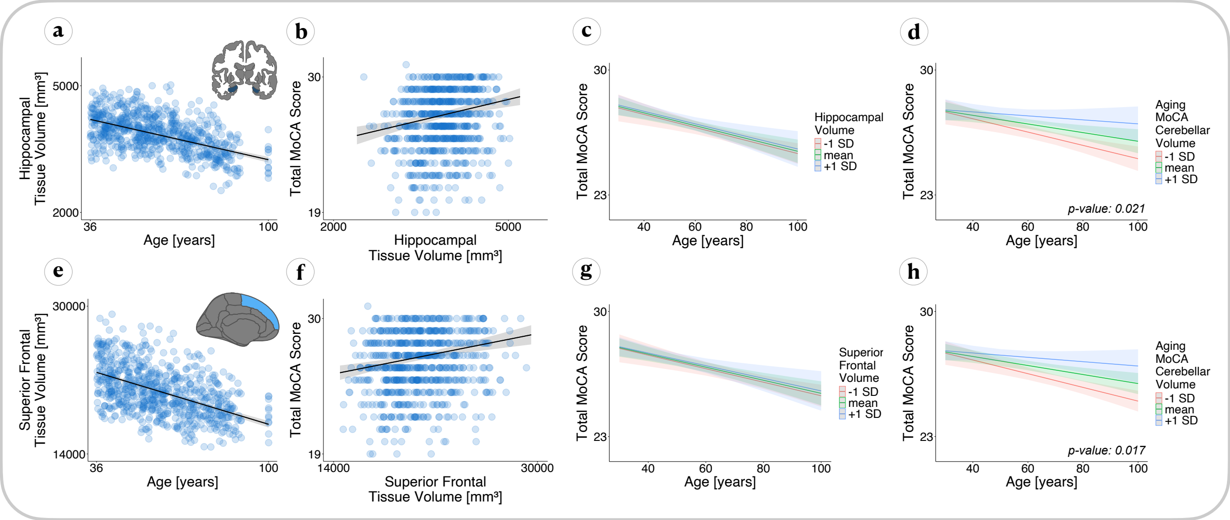

Supplementary Figure S4. Specificity analyses with hippocampus and superior frontal cortex. Relationships among age, regional tissue volume, and cognitive performance (MoCA) are shown for the hippocampus (**a-d**) and superior frontal cortex (**e-h**). Analyses were conducted in n=707 independent participants, with the individual participant serving as the unit of analysis. Points represent individual participants, solid lines denote ordinary least squares (OLS) regression fits, and shaded bands indicate 95% confidence intervals (CIs) from linear models including biological sex, estimated intracranial volume (eTIV), and years of education as covariates. In the hippocampus, (**a**) tissue volume was negatively associated with age (β=-14.91, $p<2.0\text{x}10^{-16}$) and (**b**) positively associated with MoCA (β=0.001, p=0.00001). (**c**) There was no evidence that hippocampal volume moderates the association between age and MoCA (interaction: p = 0.906). When hippocampal volume was included alongside cerebellar volume in the model (**d**), the Cerebellar Volume × Age interaction remained significant (p=0.021). In the superior frontal cortex, (**e**) tissue volume was negatively associated with age (β=-87.82, $p<2.0\text{x}10^{-16}$), and (**f**) positively associated with MoCA (β=0.0002, p=0.00001). (**g**) There was no evidence that superior frontal volume moderates the association between age and MoCA (interaction: p=0.831). (**h**) When superior frontal volume was included alongside cerebellar volume, the Cerebellar Volume × Age term remained significant (p=0.017). All statistical tests were two-sided.

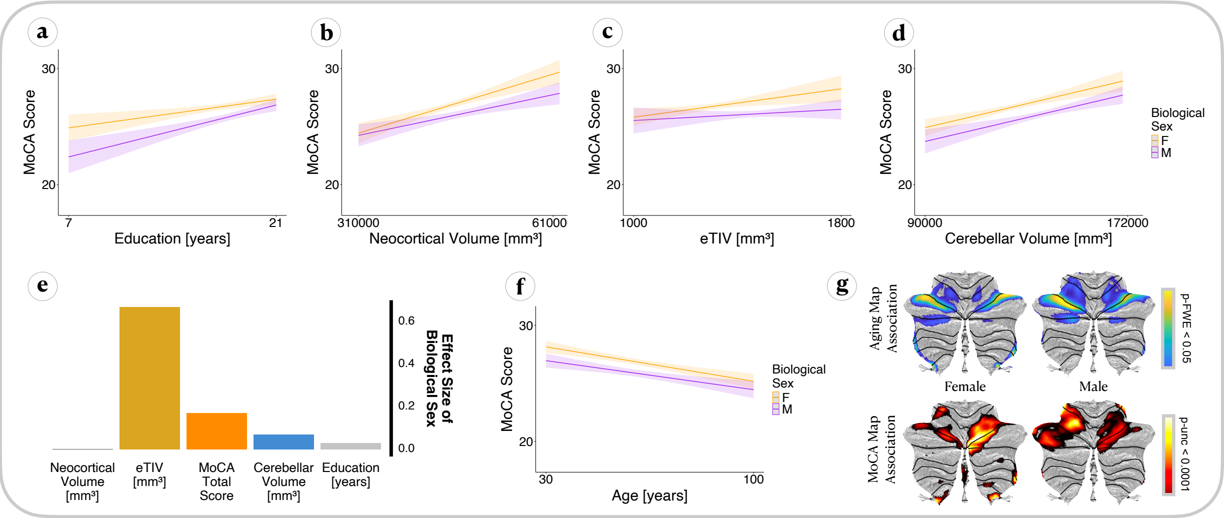

Supplementary Figure S5. Sex differences in relationships among cerebellar, cortical, and cognitive measures. Analyses were conducted in n=707 independent participants, with the individual participant serving as the unit of analysis. (**a-d**) Biological sex-stratified regression fits relating MoCA total score to years of education, neocortical volume, estimated intracranial volume (eTIV), and cerebellar volume derived from linear models including sex-by-predictor interaction terms and correcting for years of education and eTIV where appropriate. Females are shown in orange and males in purple. Solid lines represent ordinary least squares (OLS) regression fits and shaded bands indicate 95% confidence intervals (CIs). No significant sex × predictor interactions were observed for (**a**) years of education (p=0.093), (**b**) neocortical volume (p=0.161), (**c**) eTIV (p=0.274), or (**d**) cerebellar volume (p=0.912). (**e**) Marginal effect sizes (Cohen's f) for the main effect of sex across models relating sex to predictors of interest, correcting for years of education and eTIV. (**f**) Age-related differences in MoCA scores stratified by sex, showing parallel slopes across the adult lifespan, with no evidence of an Age × Sex interaction (p=0.563). (**g**) Voxel-based morphometry (VBM) maps of cerebellar gray matter associations with age (top) and MoCA total score (bottom), correcting for eTIV and shown separately for females and males. Age-related effects were broadly similar across sexes and survived family-wise error (FWE) correction (p<0.05). The MoCA association map did not survive multiple-comparisons correction and is shown uncorrected at p<0.0001 for visualization. Collectively, these analyses indicate largely comparable cerebellar and cortical correlates of cognition and aging between sexes, with only minor regional or scaling differences. All statistical tests were two-sided.

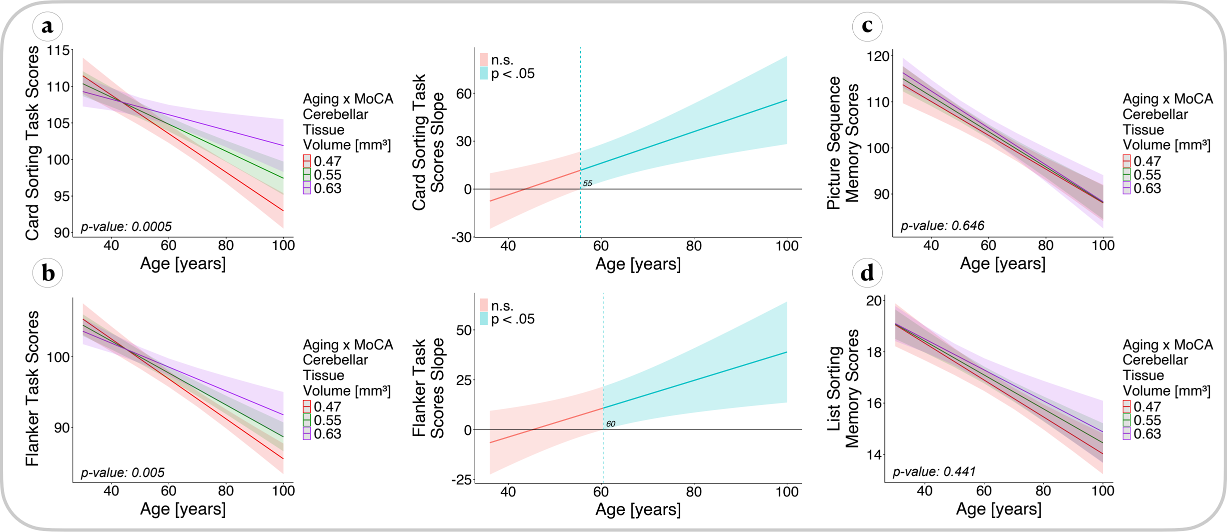

Supplementary Figure S6. Cerebellar MoCA-related volumetric signature moderates age-related performance in a separate subset of tasks. Analyses were conducted with the individual participant as the unit of analysis. Panels show relationships between age and task performance stratified by levels of the MoCA-related cerebellar volumetric signature derived from linear regression models controlling for biological sex, estimated intracranial volume (eTIV), and years of education. Solid lines represent model-estimated fits and bands indicate 95% confidence intervals (CIs). (**a-b**) Significant Age × Cerebellar Volume interactions were observed for the Dimensional Change Card Sorting task (n=605, p=0.0005) (**a: left**), and the Flanker Attention Task (n=605, p=0.005) (**b: left**). Simple slopes analysis with FDR correction (pFDR<0.05) indicated that the association between cerebellar volume and task performance became more pronounced with increasing age, with significant effects emerging after approximately 55 years for Card Sorting (**a: right**), and 60 years for Flanker Task performance (**b: right**). No evidence of Age × Cerebellar Volume interactions was observed for the (**c**) Picture Memory Sequence Task (n=605) or the (**d**) List Sorting Working Memory Task (n=620) (both p>0.4). All statistical tests were two-sided.

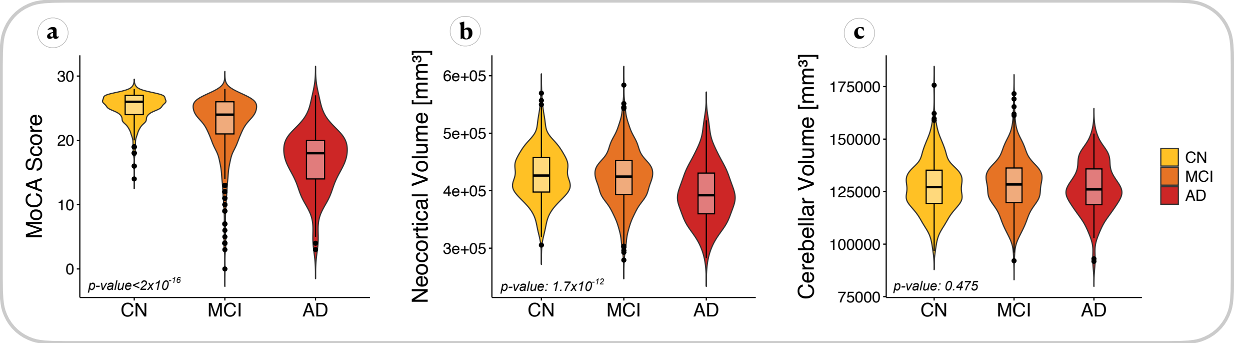

Supplementary Figure S7. MoCA scores and neocortical volume decrease with clinical diagnosis in the ADNI cohort. Distributions are shown for clinically normal (CN), mild cognitive impairment (MCI), and Alzheimer's disease (AD) participants. The unit of analysis was the individual participant. (**a**) MoCA scores differed significantly by diagnosis (one-way ANOVA, $F(2,1406)=289.60$, $p<2\times10^{-16}$). Turkey HSD post-hoc tests indicated that all three groups differed significantly from one another (mean MoCA score in CN: 25.07; MCI: 22.36; AD: 16.99). (**b**) Neocortical volume also showed a significant effect of diagnosis (one-way ANOVA, $F(2,1347)=27.68$, $p=1.7\times10^{-12}$). Post-hoc tests indicated significant differences between AD vs. MCI, and AD vs. CN, but not between CN and MCI (mean cortex volume in CN: 427,996.10 mm^3; MCI: 423,554.40 mm^3; AD: 395,070.80 mm^3). (**c**) Cerebellar volume did not differ significantly across diagnostic groups (one-way ANOVA, $F(2,1347)=0.74$, $p=0.475$), (mean cerebellar volume in CN: 127,668.20 mm^3; MCI: 128,135.30 mm^3; AD: 126,777.90 mm^3). Violin plots show kernel density distributions, with boxplots indicating the median and interquartile range and points representing individual participants. All statistical tests were two-sided.

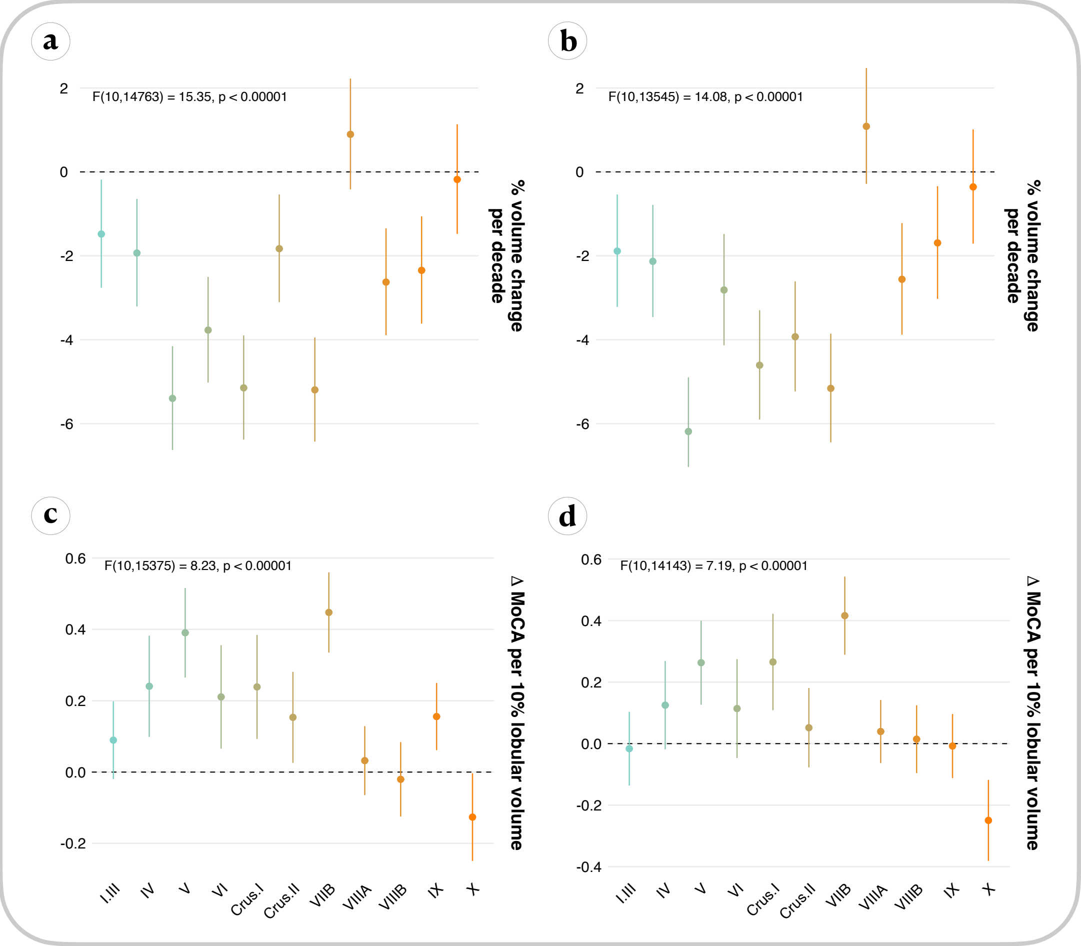

Supplementary Figure S8. Lobule-specific age and cognition associations in ADNI stratified by amyloid status. Analyses were conducted in ADNI (n=1345) participants with available amyloid status, with the individual participant as the unit of analysis. (**a-b**) Percent change in log-transformed cerebellar volume per decade (% per decade) estimated from linear models: log(volume) ~ age × lobule + sex + eTIV + hemisphere, shown separately for Aβ- (**a**) and Aβ+ (**b**) participants. Omnibus Age × Lobule effects were significant in both strata, indicating heterogeneous age-related slopes across cerebellar lobules. Lobules V, VI, crus I/II, VIIb-IX exhibited more negative age-related slopes than anterior regions (I-IV). Many posterior lobular slopes were significant after Benjamini-Hochberg (BH) correction within each stratum (Supplementary Table S6). Points denote model-estimated slopes, and vertical bars represent pointwise 95% confidence intervals (CIs). (**c-d**) Change in MoCA per 10% increase in lobular volume (mean ± pointwise 95% CIs) estimated from models: MoCA ~ log(volume) × lobule + sex + eTIV + hemisphere, shown separately for Aβ- (**c**) and Aβ+ (**d**) participants. Points denote model-estimated effects, and vertical bars indicate 95% CIs. The MoCA × Lobule interaction was significant in both strata (Aβ-: F(10,15375=8.23, p<0.00001; Aβ+: F(10,14143)=7.19, p<0.00001). Several lobules showed positive associations between volume and MoCA, particularly lobule V, crus I, and VIIb, with effects typically ranging from 0.2–0.6 MoCA points per 10% increase in volume, whereas other lobules showed smaller or near-zero associations. Lobule X exhibited a small negative association in the Aβ+ group. Full statistics and BH-adjusted tests are reported in Supplementary Table S7. All statistical tests were two-sided.

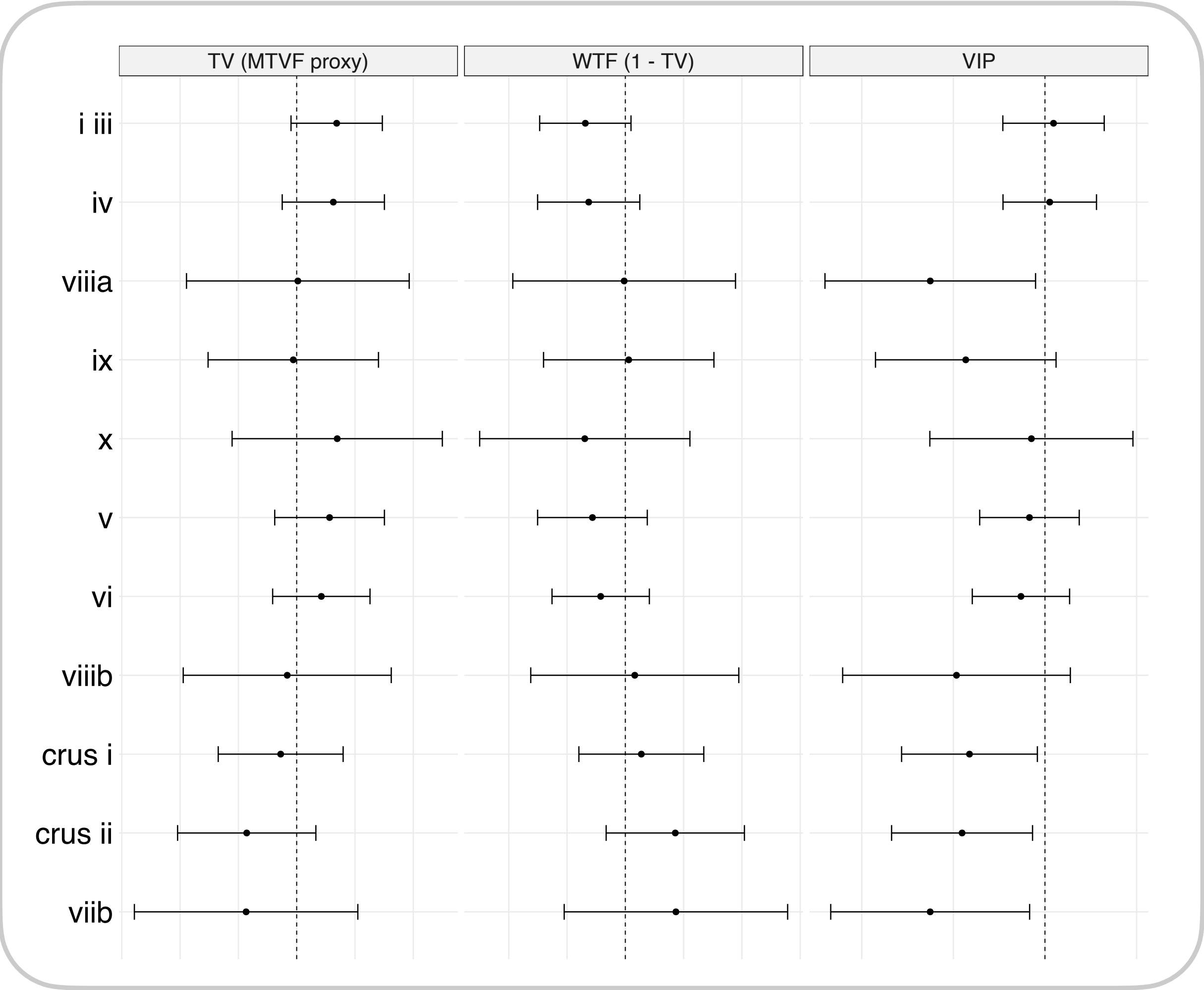

Supplementary Figure S9. Age associations for qMRI-derived tissue and microstructural metrics. Points show age regression coefficients from lobule-wise linear models fit separately for tissue volume fraction (TV; MTVF proxy), water tissue fraction (WTF = 1 – TV), and volume of interacting protons (VIP). Error bars denote 95% confidence intervals (CIs) of Age, and the dashed vertical line indicates no age association. For each participant, values were computed as the bilateral mean across left and right hemispheres for each lobule, and models included age and biological sex as predictors. These analyses were conducted in n=23 independent participants, with the individual participant as the unit of analysis. The pattern of VIP-age associations occurs alongside related, but not identical, age associations for TV and WTF, consistent with VIP capturing microstructural variation not reducible to tissue fraction or water fraction alone. All statistical tests were two-sided.

Supplementary Tables

Table S1. Estimated age-related volume change per cerebellar lobule

Lobule	% Change per Decade [95% CI]	*p*-BH
I-III	-1.30% [-1.88, -0.72]	0.00002
IV	-1.65% [-2.22, -1.07]	<0.00001
V	-4.98% [-5.54, -4.43]	<0.00001
VI	-0.86% [-1.44, -0.28]	0.005
Crus I	-3.74% [-4.30, -3.17]	<0.00001
Crus II	-2.63% [-3.19, -2.05]	<0.00001
VIIb	-3.37% [-3.94, -2.81]	<0.00001
VIIIa	0.48% [-0.10, 1.07]	0.108
VIIIb	-2.91% [-3.47, -2.34]	<0.00001
IX	-3.05% [-3.62, -2.48]	<0.00001
X	0.49% [-0.10, 1.07]	0.108

Notes: Estimated age-related volume change per cerebellar lobule. Slopes reflect percentage change in lobular volume, derived from log-transformed volume models (log(volume) ~ age × lobule + sex + eTIV + hemisphere + years of education). Values are mean estimates ±95% confidence intervals (z-based). P-values are Benjamini-Hochberg (BH) corrected across lobules.

Table S2. Estimated age-related volume change per cerebellar vermis subregion

Lobule	% Change per Decade [95% CI]	*p*-BH
Vermis VI	-1.56% [-2.26, -0.86]	0.00008
Vermis VII	-1.31% [-2.02, -0.61]	0.0007
Vermis VIII	0.19% [-0.52, 0.91]	0.593
Vermis IX	-0.99% [-1.69, -0.28]	0.008
Vermis X	-1.09% [-1.80, -0.39]	0.004

Notes: Estimated age-related volume change per cerebellar vermis region. Slopes reflect percentage change in vermis volume, derived from log-transformed volume models (log(volume) ~ age × vermis + sex + eTIV + hemisphere + years of education). Values are mean estimates ±95% confidence intervals (z-based). P-values are Benjamini-Hochberg (BH) corrected across lobules.

Table S3. Estimated age-related T1w/T2w change per cerebellar lobule

Lobule	% Change per Decade [95% CI]	*p*-BH
I-III	-0.84% [-1.24, -0.43]	0.0001
IV	-1.58% [-1.98, -1.17]	<0.00001
V	-1.78% [-2.18, -1.37]	<0.00001
VI	-1.99% [-2.39, -1.59]	<0.00001
Crus I	-3.13% [-3.53, -2.74]	<0.00001
Crus II	-2.98% [-3.38, -2.58]	<0.00001
VIIb	-2.54% [-2.94, -2.14]	<0.00001
VIIIa	-2.41% [-2.81, -2.01]	<0.00001
VIIIb	-2.84% [-3.24, -2.44]	<0.00001
IX	-3.14% [-3.53, -2.74]	<0.00001
X	-1.91% [-2.31, -1.50]	<0.00001

Notes: Estimated age-related T1w/T2w change per cerebellar lobule. Slopes reflect percentage change in lobular T1w/T2w ratio, derived from log-transformed volume models (log(T1w/T2w) ~ age × lobule + sex + eTIV + hemisphere + years of education). Values are mean estimates ±95% confidence intervals (z-based). P-values are Benjamini-Hochberg (BH) corrected across lobules.

Table S4. Estimated age-related voxel-wise intracellular proton density change per cerebellar lobule

Lobule	% Change per Decade [95% CI]	*p*-value	*p*-BH
I-III	0.25% [-1.51, 2.05]	0.755	0.852
IV	0.16% [-1.60, 1.95]	0.859	0.859
V	-0.39% [-2.14, 1.39]	0.661	0.829
VI	-0.62% [-2.36, 1.16]	0.485	0.763
Crus I	-1.96% [-3.69, -0.21]	0.029	0.053
Crus II	-2.01% [-3.73, -0.26]	0.025	0.053
VIIb	-2.76% [-4.47, -1.02]	0.003	0.015
VIIIa	-2.81% [-4.51, -1.07]	0.002	0.015
VIIIb	-2.10% [-3.82, -0.35]	0.020	0.053
IX	-2.01% [-3.73, -0.26]	0.026	0.053
X	-0.37% [-2.12, -1.42]	0.678	0.829

Notes: Estimated age-related voxel-wise intracellular proton (VIP) density change per cerebellar lobule. Slopes reflect percentage change in lobular T1w/T2w ratio, derived from log-transformed volume models (log(VIP) ~ age × lobule + sex + hemisphere). Values are mean estimates ±95% confidence intervals. P-values are Benjamini-Hochberg (BH) corrected across lobules.

Table S5. Estimated change in MoCA per 10% lobule volume in HCP-Aging

Lobule	MoCA Change per 10% Volume [95% CI]	*p*-BH
I-III	0.05% [-0.02, 0.11]	0.139
IV	0.13% [0.05, 0.20]	0.002
V	0.18% [0.12, 0.25]	<0.00001
VI	0.27% [0.19, 0.35]	<0.00001
Crus I	0.27% [0.19, 0.35]	<0.00001
Crus II	0.21% [0.14, 0.28]	<0.00001
VIIb	0.15% [0.08, 0.22]	0.00004
VIIIa	0.06% [-0.001, 0.12]	0.061
VIIIb	0.15% [0.09, 0.21]	<0.00001
IX	0.16% [0.11, 0.22]	<0.00001
X	0.11% [0.04, 0.18]	0.004

Notes: Estimated change in MoCA per 10% lobule volume in HCP-Aging. Slopes reflect percentage change in MoCA score, derived from log-transformed volume models (MoCA ~ log(volume) × lobule + sex + eTIV + years of education + hemisphere). Values are mean estimates ±95% confidence intervals (*t*-based). P-values are Benjamini-Hochberg (BH) corrected across lobules.

Table S6. Cerebellar Volume × Diagnosis Aβ Group

	Dependent variable:
	**MoCA Score**
Constant	(18.987, 27.844)<br>t = 10.363<br>p = 0.000***
Biological Sex	(-0.693, 0.222)<br>t = -1.007<br>p = 0.314
eTIV	(-0.00000, 0.00000)<br>t = -0.759<br>p = 0.449
Total Cerebellar Volume	(-0.00001, 0.0001)<br>t = 1.135<br>p = 0.257
CN Aβ+	(-7.409, 4.967)<br>t = -0.387<br>p = 0.700
MCI Aβ-	(-10.183, 1.169)<br>t = -1.556<br>p = 0.120
MCI Aβ+	(-10.964, 0.906)<br>t = -1.661<br>p = 0.097
AD Aβ-	(-32.022, -12.609)<br>t = -4.506<br>p = 0.00001***
AD Aβ+	(-21.419, -0.123)<br>t = -1.983<br>p = 0.048*
Cerebellar Volume × CN Aβ+	(-0.00004, 0.0001)<br>t = 0.469<br>p = 0.639
Cerebellar Volume × MCI Aβ-	(-0.00003, 0.0001)<br>t = 0.742<br>p = 0.459
Cerebellar Volume × MCI Aβ+	(-0.00003, 0.0001)<br>t = 0.867<br>p = 0.386
Cerebellar Volume × AD Aβ-	(0.00004, 0.0002)

	t = 2.909<br>p = 0.004**
Cerebellar Volume × AD Aβ+	(-0.0001, 0.0001)<br>t = 0.597<br>p = 0.551
Observations	1,344
R^2	0.311
Adjusted R^2	0.304
Residual Std. Error	3.534 (df = 1330)
F Statistic	46.167*** (df = 13; 1330)

Note: *p<0.05, **p<0.01, ***p<0.001

Data is reported as mean (standard deviation).
Abbreviations: estimated total intracranial volume, eTIV; clinically normal, CN; mild cognitive impairment, MCI; Alzheimer's disease, AD; amyloid-negative, Aβ-; amyloid-positive, Aβ+.

Table S7. Estimated age-related volume change per cerebellar lobule in ADNI

	Aβ-		Aβ+	
Lobule	% Change per Decade [95% CI]	*p*-BH	% Change per Decade [95% CI]	*p*-BH
I-III	-1.48% [-2.76, -0.18]	0.031	-1.89% [-3.22, -0.54]	0.009
IV	-1.93% [-3.21, -0.64]	0.005	-2.13% [-3.46, -0.79]	0.003
V	-5.40% [-6.63, -4.15]	<0.00001	-6.19% [-7.46, -4.90]	<0.00001
VI	-3.77% [-5.02, -2.50]	<0.00001	-2.81% [-4.13, -1.48]	0.0001
Crus I	-5.15% [-6.38, -3.90]	<0.00001	-4.61% [-5.90, -3.30]	<0.00001
Crus II	-1.83% [-3.11, -0.54]	0.008	-3.93% [-5.23, -2.61]	<0.00001
VIIb	-5.20% [-6.43, -3.95]	<0.00001	-5.16% [-6.45, -3.86]	<0.00001
VIIIa	0.90% [-0.42, 2.22]	0.200	1.09% [-0.29, 2.48]	0.133
VIIIb	-2.63% [-3.89, -1.34]	0.0001	-2.56% [-3.88, -1.22]	0.0004
IX	-2.35% [-3.62, -1.06]	0.0007	-1.69% [-3.03, -0.34]	0.0174
X	-0.18% [-1.48, 1.13]	0.787	-0.36% [-1.71, 1.01]	0.607

Notes: Estimated age-related volume change per cerebellar lobule in ADNI. Slopes reflect percentage change in lobular volume, derived from log-transformed volume models (log(volume) ~ age × lobule + sex + eTIV + hemisphere). Values are mean estimates ±95% confidence intervals (*t*-based). P-values are Benjamini-Hochberg (BH) corrected across lobules.

Table S8. Estimated change in MoCA per 10% lobule volume in ADNI

	Aβ-		Aβ+	
Lobule	MoCA Change per 10% Volume [95% CI]	*p*-BH	MoCA Change per 10% Volume [95% CI]	*p*-BH
I-III	0.09% [-0.02, 0.20]	0.131	-0.02% [-0.14, 0.10]	0.876
IV	0.24% [0.10, 0.38]	0.003	0.13% [-0.02, 0.27]	0.194
V	0.39% [0.27, 0.52]	<0.00001	0.26% [0.13, 0.40]	0.0008
VI	0.21% [0.07, 0.36]	0.008	0.11% [-0.05, 0.28]	0.301
Crus I	0.24% [0.09, 0.39]	0.003	0.27% [0.11, 0.42]	0.003
Crus II	0.15% [0.03, 0.28]	0.029	0.05% [-0.08, 0.18]	0.620
VIIb	0.45% [0.34, 0.56]	<0.00001	0.42% [0.29, 0.54]	<0.00001
VIIIa	0.03% [-0.07, 0.13]	0.566	0.04% [-0.06, 0.14]	0.620
VIIIb	-0.02% [-0.12, 0.08]	0.704	0.01% [-0.10, 0.13]	0.876
IX	0.16% [0.06, 0.25]	0.003	-0.01% [-0.11, 0.10]	0.883
X	-0.13% [-0.25, -0.004]	0.060	-0.25% [-0.38, -0.12]	0.001

Notes: Estimated change in MoCA per 10% lobule volume in ADNI. Slopes reflect percentage change in MoCA score, derived from log-transformed volume models (MoCA ~ log(volume) × lobule + sex + eTIV + hemisphere). Values are mean estimates ±95% confidence intervals (*t*-based). P-values are Benjamini-Hochberg (BH) corrected across lobules.

Table S9. ADNI T1-Weighted MRI Protocols

ADNI Phase	Field Strength	Sequence	TR (ms)	TE (ms)	TI (ms)	Flip Angle	Voxel Size (mm^3)	Scan Time
ADNI-1	1.5 Tesla (with ~25% also at 3T)	3D T1 MPRAGE	2400	3.0 min full echo	1000	8°	~1.2mm^3 (256x256x170 matrix)	~9-10 min (two acquisitions back-to-back)
ADNI-GO/2	3 Tesla	3D T1 MPRAGE / IR-SPGR	2300	2.8-3.1 min full echo	900	8°	1mm^3 (256x256x170 matrix)	~6 min (two acquisitions back-to-back; full vs accelerated)
ADNI-3	3 Tesla	3D T1 MPRAGE / IR-SPGR	2300	2.8-3.1 min full echo	900	8°	1mm^3 (208x240x256 matrix)	~6 min (2x accelerated image acquisition)

Notes: ADNI scan protocols were vendor-specific but harmonized to produce equivalent images across GE, Siemens, and Philips platforms. All sites followed a standardized protocol established by the ADNI MRI Core to ensure consistent image contrast and resolution across platforms. Settings provided high-resolution T1-weighted images suitable for volumetric analysis with careful calibration to ensure equivalent image quality across manufacturers. "*min full TE*" refers to the minimum full echo time achievable (varies slightly by vendor; approximately 2.8–3.5 ms). The ADNI quality control center (Mayo Clinic) monitored all uploads and checked sequence parameters to ensure compliance; any deviations required a rescan (e.g., head coverage, SNR, artifacts). As a result, the collected 3T T1 images are directly comparable. A phantom scan (ADNI agar phantom) was run at each session after human scans, using an accelerated MPRAGE with the same settings, to monitor scanner stability. Abbreviations: Alzheimer's Disease Neuroimaging Initiative (ADNI), inversion recovery-prepared spoiled gradient recalled echo (IR-SPGR), magnetization prepared rapid gradient echo (MPRAGE), millisecond (ms), echo time (TE), inversion time (TI), repetition time (TR).
