# Supplementary material for: Cerebellar aging is spatially heterogeneous and supports cognitive resilience in later life
Source: Nat Neurosci. 2026 Jun 10;29(7):1699–710. doi: 10.1038/s41593-026-02289-x (PMC13337503; doi:10.1038/s41593-026-02289-x)
Supplement: Supplementary file 2 — Reporting Summary [file 41593_2026_2289_MOESM2_ESM.pdf]

nature portfolio

Corresponding author(s): uquillas@princeton.edu

Last updated by author(s): 03/10/2026

Reporting Summary

Nature Portfolio wishes to improve the reproducibility of the work that we publish. This form provides structure for consistency and transparency in reporting. For further information on Nature Portfolio policies, see our Editorial Policies and the Editorial Policy Checklist.

Statistics

For all statistical analyses, confirm that the following items are present in the figure legend, table legend, main text, or Methods section.

n/a	Confirmed	
☐	☒	The exact sample size (*n*) for each experimental group/condition, given as a discrete number and unit of measurement
☐	☒	A statement on whether measurements were taken from distinct samples or whether the same sample was measured repeatedly
☐	☒	The statistical test(s) used AND whether they are one- or two-sided<br>*Only common tests should be described solely by name; describe more complex techniques in the Methods section.*
☐	☒	A description of all covariates tested
☐	☒	A description of any assumptions or corrections, such as tests of normality and adjustment for multiple comparisons
☐	☒	A full description of the statistical parameters including central tendency (e.g. means) or other basic estimates (e.g. regression coefficient) AND variation (e.g. standard deviation) or associated estimates of uncertainty (e.g. confidence intervals)
☐	☒	For null hypothesis testing, the test statistic (e.g. *F*, *t*, *r*) with confidence intervals, effect sizes, degrees of freedom and *P* value noted<br>*Give P values as exact values whenever suitable.*
☒	☐	For Bayesian analysis, information on the choice of priors and Markov chain Monte Carlo settings
☒	☐	For hierarchical and complex designs, identification of the appropriate level for tests and full reporting of outcomes
☐	☒	Estimates of effect sizes (e.g. Cohen's *d*, Pearson's *r*), indicating how they were calculated

Our web collection on statistics for biologists contains articles on many of the points above.

Software and code

Policy information about availability of computer code

Data collection	Siemens 3T Magnetom Prisma for quantitative MRI data at the Princeton Neuroscience Institute (https://github.com/mezera/mrQ)
Data analysis	FreeSurfer (7.1.1) , ACAPULCO cerebellar segmentation pipeline (Han et al., 2020), R (v.4.0.4: https:www.R-project.org), FSL (6.0.2)

For manuscripts utilizing custom algorithms or software that are central to the research but not yet described in published literature, software must be made available to editors and reviewers. We strongly encourage code deposition in a community repository (e.g. GitHub). See the Nature Portfolio guidelines for submitting code & software for further information.

Data

Policy information about availability of data

All manuscripts must include a data availability statement. This statement should provide the following information, where applicable:

- Accession codes, unique identifiers, or web links for publicly available datasets
- A description of any restrictions on data availability
- For clinical datasets or third party data, please ensure that the statement adheres to our policy

Data analyzed are available at public repositories, which may require appropriate data sharing agreements from each individual publicly accessible database, including LONI for ADNI and HCP data (https://ida.loni.usc.edu/), and biobankUK for UK Biobank data (https://biobank.ctsu.ox.ac.uk). Deidentified source data for main figures can be found at:

https://github.com/fdoleireuquillas/AgingCerebellum. Source code related to statistical analysis and visualization can be found at: https://github.com/fdoleireuquillas/AgingCerebellum.

Research involving human participants, their data, or biological material

Policy information about studies with human participants or human data. See also policy information about sex, gender (identity/presentation), and sexual orientation and race, ethnicity and racism.

Reporting on sex and gender	In HCP-Aging, Female = 400 (56.5%) (Table 1a); in UK Biobank, 52.9% were female (Table 1b); and ADNI sex counts are provided for each diagnostic/amyloid cell (Table 1c). Clinically Normal Amyloid Negative, Female = 55.4%; Clinically Normal Amyloid Positive, Female = 56.1%; Mild Cognitive Impairment Amyloid Negative, Female = 40%; Mild Cognitive Impairment Amyloid Positive, Female = 36.6%; Alzheimer's Disease Dementia Amyloid Negative, Female = 37.5; Alzheimer's Disease Dementia Amyloid Positive, Female = 45.2%. All participants provided informed consent, and each cohort adhered to institutional review board guidelines.
Reporting on race, ethnicity, or other socially relevant groupings	Demographics<br>We included a racially diverse sample of participants with complete cerebellar coverage on MRI scans, as well as corresponding cognitive testing. In the HCP-Aging cohort, there were 2 (0.28%) American Indian/Alaskan Native individuals, 52 (7.20%) Asian, 98 (13.84%) Black or African American, 506 (71.61%) White, 36 (4.38%) more than one race, and 14 (1.98%) individuals with unknown or unreported race, with 69 (9.75%) identifying as Hispanic or Latino, 632 (89.27%) not Hispanic or Latino, and 7 (0.28%) of unreported ethnic group. We also sourced MRI data from the UK Biobank, but did not have access to ethnic or racial status. Additionally, our ADNI cohort comprised of 2 (0.14%) American Indian or Alaskan Native individuals, 7 (0.50%) Asian, 22 (1.55%) Black or African American, 1 (0.07%) Native Hawaiian or other Pacific Islander, 670 (47.08%) White, 11 (0.77%) individuals of more than one race, and 710 (49.89%) individuals of unknown or unreported racial status, with 16 (1.12%) identifying as Hispanic or Latino, 694 (48.77%) not Hispanic or Latino, and 713 (50.11%) of unreported ethnic group.
Population characteristics	The HCP-Aging cohort included 708 participants aged 36–100 years. The UK Biobank dataset provided 45,013 participants aged 44–81 years, and the ADNI cohort included 1,423 participants aged 56–95 years old.
Recruitment	Human Connectome Project-Aging (HCP-Aging)<br>Participants were recruited as part of the Human Connectome Project in Aging, a multisite initiative supported by the NIH. Recruitment targeted cognitively healthy adults aged 36-100 years. Individuals were identified through community outreach, registries, family members of HCP participants, and institutional participant pools. Eligibility was determined through structured screening that excluded major neurological or psychiatric illness, contraindications to MRI, and significant cognitive impairment. All participants provided written informed consent under protocols approved by the Washington University in St. Louis Institutional Review Board.<br><br>UK Biobank<br>Participants were recruited through the UK National Health Service population registry. ~500,000 adults aged 40-69 years were invited between 2006-2010 via mailed invitations; those who volunteered attended one of 22 assessment centers throughout the United Kingdom. The neuroimaging subset was recruited from this broader cohort and invited back for MRI scanning based on geographic proximity to imaging centers and willingness to participate in follow-up assessments. Inclusion/exclusion criteria were minimal, consistent with UK Biobank's population-level design, but excluded individuals unable to provide informed consent. Ethical approval was obtained from the UK Biobank Research Ethics Committee, and all participants gave written informed consent.<br><br>Alzheimer's Disease Neuromaging Initiative (ADNI)<br>Participants were recruited across ~60 North American sites using a combination of clinician referral, community outreach, memory clinics, and media advertisements. ADNI enrolls individuals spanning normal aging, mild cognitive impairment (MCI), and Alzheimer's disease (AD) dementia, following prespecified diagnostic criteria based on clinical evaluation, neuropsychological testing, and functional assessment. Key exclusion criteria included significant neurological disease other than AD, major psychiatric disorders, MRI contraindications, and unstable medical illness. Participants provided written informed consent, and study procedures were approved by local institutional review boards at all participating centers.<br><br>Princeton validation sample<br>The Princeton qMRI sample was recruited locally through campus and community advertisements. Eligible participants were healthy adults with no history of neurological or major psychiatric illness and no MRI contraindications. All participants provided written informed consent under a protocol approved by the Princeton University Institutional Review Board.
Ethics oversight	All datasets received IRB/ethics approval and written informed consent (HCP-Aging, UK Biobank, ADNI, Princeton qMRI). Only de-identified data were used.

Note that full information on the approval of the study protocol must also be provided in the manuscript.

Field-specific reporting

Please select the one below that is the best fit for your research. If you are not sure, read the appropriate sections before making your selection.

☒ Life sciences ☐ Behavioural & social sciences ☐ Ecological, evolutionary & environmental sciences

For a reference copy of the document with all sections, see nature.com/documents/nr-reporting-summary-flat.pdf

Life sciences study design

All studies must disclose on these points even when the disclosure is negative.

Sample size	Sample sizes were determined by the availability of eligible participants within each cohort (HCP-Aging n=708, UK Biobank n=47,144, ADNI n=1,423, Princeton Neuroscience Institute n=23). These are large, well-established neuroimaging studies with prespecified recruitment targets and publicly defined protocols. No a priori power calculations were performed by the present study, as analyses used all participants meeting inclusion/exclusion criteria, imaging quality thresholds, and data completeness requirements.
Data exclusions	Participants were excluded only if imaging data did not meet quality-control criteria. MRI exclusions included scans with insufficient field of view (e.g., incomplete coverage of the cerebellum), substantial motion or artifact, or failed preprocessing. For analyses using ACAPULCO cerebellar parcellation, participants were additionally excluded if automated lobular segmentations did not meet visual quality-control standards. Only individuals with complete demographic data and usable MRI after QC were included.
Replication	Key findings were replicated across independent datasets and imaging modalities. Primary analyses conducted in the HCP-Aging cohort were conceptually and quantitatively replicated in the UK Biobank using analogous structural MRI measures and identical statistical models. We further replicated the spatial pattern of age-related cerebellar differences in an independent quantitative MRI dataset collected at Princeton University, demonstrating consistency across acquisition platforms and imaging modalities. All replication analyses were conducted independently and reproduced the direction and spatial distribution of the original effects.
Randomization	Participants were allocated to groups based on cohort-defined diagnostic or demographic categories rather than random assignment. Since the study was observational, randomization was not applicable. Statistical models included Sex, total cranial/brain volume, and education (when available) to control for systematic group differences.
Blinding	Blinding was not applicable because this was an observational neuroimaging study with no experimentally assigned groups. Imaging preprocessing and statistical analyses were performed using standardized, automatic pipelines. For steps requiring manual quality control (e.g., verifying cerebellar field-of-view coverage or ACAPULCO cerebellar segmentation), raters were blinded to participants' demographic and cognitive data.

Behavioural & social sciences study design

All studies must disclose on these points even when the disclosure is negative.

Study description	*Briefly describe the study type including whether data are quantitative, qualitative, or mixed-methods (e.g. qualitative cross-sectional, quantitative experimental, mixed-methods case study).*
Research sample	*State the research sample (e.g. Harvard university undergraduates, villagers in rural India) and provide relevant demographic information (e.g. age, sex) and indicate whether the sample is representative. Provide a rationale for the study sample chosen. For studies involving existing datasets, please describe the dataset and source.*
Sampling strategy	*Describe the sampling procedure (e.g. random, snowball, stratified, convenience). Describe the statistical methods that were used to predetermine sample size OR if no sample-size calculation was performed, describe how sample sizes were chosen and provide a rationale for why these sample sizes are sufficient. For qualitative data, please indicate whether data saturation was considered, and what criteria were used to decide that no further sampling was needed.*
Data collection	*Provide details about the data collection procedure, including the instruments or devices used to record the data (e.g. pen and paper, computer, eye tracker, video or audio equipment) whether anyone was present besides the participant(s) and the researcher, and whether the researcher was blind to experimental condition and/or the study hypothesis during data collection.*
Timing	*Indicate the start and stop dates of data collection. If there is a gap between collection periods, state the dates for each sample cohort.*
Data exclusions	*If no data were excluded from the analyses, state so OR if data were excluded, provide the exact number of exclusions and the rationale behind them, indicating whether exclusion criteria were pre-established.*
Non-participation	*State how many participants dropped out/declined participation and the reason(s) given OR provide response rate OR state that no participants dropped out/declined participation.*
Randomization	*If participants were not allocated into experimental groups, state so OR describe how participants were allocated to groups, and if allocation was not random, describe how covariates were controlled.*

Ecological, evolutionary & environmental sciences study design

All studies must disclose on these points even when the disclosure is negative.

Study description	*Briefly describe the study. For quantitative data include treatment factors and interactions, design structure (e.g. factorial, nested, hierarchical), nature and number of experimental units and replicates.*
Research sample	*Describe the research sample (e.g. a group of tagged Passer domesticus, all Stenocereus thurberi within Organ Pipe Cactus National*

Research sample	*Monument), and provide a rationale for the sample choice. When relevant, describe the organism taxa, source, sex, age range and any manipulations. State what population the sample is meant to represent when applicable. For studies involving existing datasets, describe the data and its source.*
Sampling strategy	*Note the sampling procedure. Describe the statistical methods that were used to predetermine sample size OR if no sample-size calculation was performed, describe how sample sizes were chosen and provide a rationale for why these sample sizes are sufficient.*
Data collection	*Describe the data collection procedure, including who recorded the data and how.*
Timing and spatial scale	*Indicate the start and stop dates of data collection, noting the frequency and periodicity of sampling and providing a rationale for these choices. If there is a gap between collection periods, state the dates for each sample cohort. Specify the spatial scale from which the data are taken*
Data exclusions	*If no data were excluded from the analyses, state so OR if data were excluded, describe the exclusions and the rationale behind them, indicating whether exclusion criteria were pre-established.*
Reproducibility	*Describe the measures taken to verify the reproducibility of experimental findings. For each experiment, note whether any attempts to repeat the experiment failed OR state that all attempts to repeat the experiment were successful.*
Randomization	*Describe how samples/organisms/participants were allocated into groups. If allocation was not random, describe how covariates were controlled. If this is not relevant to your study, explain why.*
Blinding	*Describe the extent of blinding used during data acquisition and analysis. If blinding was not possible, describe why OR explain why blinding was not relevant to your study.*

Did the study involve field work? ☐ Yes ☐ No

Field work, collection and transport

Field conditions	*Describe the study conditions for field work, providing relevant parameters (e.g. temperature, rainfall).*
Location	*State the location of the sampling or experiment, providing relevant parameters (e.g. latitude and longitude, elevation, water depth).*
Access & import/export	*Describe the efforts you have made to access habitats and to collect and import/export your samples in a responsible manner and in compliance with local, national and international laws, noting any permits that were obtained (give the name of the issuing authority, the date of issue, and any identifying information).*
Disturbance	*Describe any disturbance caused by the study and how it was minimized.*

Reporting for specific materials, systems and methods

We require information from authors about some types of materials, experimental systems and methods used in many studies. Here, indicate whether each material, system or method listed is relevant to your study. If you are not sure if a list item applies to your research, read the appropriate section before selecting a response.

Materials & experimental systems

n/a	Involved in the study	
☒	☐	Antibodies
☒	☐	Eukaryotic cell lines
☒	☐	Palaeontology and archaeology
☒	☐	Animals and other organisms
☒	☐	Clinical data
☒	☐	Dual use research of concern
☒	☐	Plants

Methods

n/a	Involved in the study	
☒	☐	ChIP-seq
☒	☐	Flow cytometry
☒	☐	MRI-based neuroimaging

Antibodies

Antibodies used	*Describe all antibodies used in the study; as applicable, provide supplier name, catalog number, clone name, and lot number.*
Validation	*Describe the validation of each primary antibody for the species and application, noting any validation statements on the manufacturer's website, relevant citations, antibody profiles in online databases, or data provided in the manuscript.*

Eukaryotic cell lines

Policy information about cell lines and Sex and Gender in Research

Cell line source(s)	*State the source of each cell line used and the sex of all primary cell lines and cells derived from human participants or vertebrate models.*
Authentication	*Describe the authentication procedures for each cell line used OR declare that none of the cell lines used were authenticated.*
Mycoplasma contamination	*Confirm that all cell lines tested negative for mycoplasma contamination OR describe the results of the testing for mycoplasma contamination OR declare that the cell lines were not tested for mycoplasma contamination.*
Commonly misidentified lines (See ICLAC register)	*Name any commonly misidentified cell lines used in the study and provide a rationale for their use.*

Palaeontology and Archaeology

Specimen provenance	*Provide provenance information for specimens and describe permits that were obtained for the work (including the name of the issuing authority, the date of issue, and any identifying information). Permits should encompass collection and, where applicable, export.*
Specimen deposition	*Indicate where the specimens have been deposited to permit free access by other researchers.*
Dating methods	*If new dates are provided, describe how they were obtained (e.g. collection, storage, sample pretreatment and measurement), where they were obtained (i.e. lab name), the calibration program and the protocol for quality assurance OR state that no new dates are provided.*

☐ Tick this box to confirm that the raw and calibrated dates are available in the paper or in Supplementary Information.

Ethics oversight	*Identify the organization(s) that approved or provided guidance on the study protocol, OR state that no ethical approval or guidance was required and explain why not.*

Note that full information on the approval of the study protocol must also be provided in the manuscript.

Animals and other research organisms

Policy information about studies involving animals; ARRIVE guidelines recommended for reporting animal research, and Sex and Gender in Research

Laboratory animals	*For laboratory animals, report species, strain and age OR state that the study did not involve laboratory animals.*
Wild animals	*Provide details on animals observed in or captured in the field; report species and age where possible. Describe how animals were caught and transported and what happened to captive animals after the study (if killed, explain why and describe method; if released, say where and when) OR state that the study did not involve wild animals.*
Reporting on sex	*Indicate if findings apply to only one sex; describe whether sex was considered in study design, methods used for assigning sex. Provide data disaggregated for sex where this information has been collected in the source data as appropriate; provide overall numbers in this Reporting Summary. Please state if this information has not been collected. Report sex-based analyses where performed, justify reasons for lack of sex-based analysis.*
Field-collected samples	*For laboratory work with field-collected samples, describe all relevant parameters such as housing, maintenance, temperature, photoperiod and end-of-experiment protocol OR state that the study did not involve samples collected from the field.*
Ethics oversight	*Identify the organization(s) that approved or provided guidance on the study protocol, OR state that no ethical approval or guidance was required and explain why not.*

Note that full information on the approval of the study protocol must also be provided in the manuscript.

Clinical data

Policy information about clinical studies

All manuscripts should comply with the ICMJE guidelines for publication of clinical research and a completed CONSORT checklist must be included with all submissions.

Clinical trial registration	*Provide the trial registration number from ClinicalTrials.gov or an equivalent agency.*
Study protocol	*Note where the full trial protocol can be accessed OR if not available, explain why.*
Data collection	*Describe the settings and locales of data collection, noting the time periods of recruitment and data collection.*

Outcomes	*Describe how you pre-defined primary and secondary outcome measures and how you assessed these measures.*

Dual use research of concern

Policy information about dual use research of concern

Hazards

Could the accidental, deliberate or reckless misuse of agents or technologies generated in the work, or the application of information presented in the manuscript, pose a threat to:

No	Yes	
☐	☐	Public health
☐	☐	National security
☐	☐	Crops and/or livestock
☐	☐	Ecosystems
☐	☐	Any other significant area

Experiments of concern

Does the work involve any of these experiments of concern:

No	Yes	
☐	☐	Demonstrate how to render a vaccine ineffective
☐	☐	Confer resistance to therapeutically useful antibiotics or antiviral agents
☐	☐	Enhance the virulence of a pathogen or render a nonpathogen virulent
☐	☐	Increase transmissibility of a pathogen
☐	☐	Alter the host range of a pathogen
☐	☐	Enable evasion of diagnostic/detection modalities
☐	☐	Enable the weaponization of a biological agent or toxin
☐	☐	Any other potentially harmful combination of experiments and agents

Plants

Seed stocks	*Report on the source of all seed stocks or other plant material used. If applicable, state the seed stock centre and catalogue number. If plant specimens were collected from the field, describe the collection location, date and sampling procedures.*
Novel plant genotypes	*Describe the methods by which all novel plant genotypes were produced. This includes those generated by transgenic approaches, gene editing, chemical/radiation-based mutagenesis and hybridization. For transgenic lines, describe the transformation method, the number of independent lines analyzed and the generation upon which experiments were performed. For gene-edited lines, describe the editor used, the endogenous sequence targeted for editing, the targeting guide RNA sequence (if applicable) and how the editor was applied.*
Authentication	*Describe any authentication procedures for each seed stock used or novel genotype generated. Describe any experiments used to assess the effect of a mutation and, where applicable, how potential secondary effects (e.g. second site T-DNA insertions, mosiacism, off-target gene editing) were examined.*

ChIP-seq

Data deposition

☐ Confirm that both raw and final processed data have been deposited in a public database such as GEO.

☐ Confirm that you have deposited or provided access to graph files (e.g. BED files) for the called peaks.

Data access links<br>*May remain private before publication.*	*For "Initial submission" or "Revised version" documents, provide reviewer access links. For your "Final submission" document, provide a link to the deposited data.*
Files in database submission	*Provide a list of all files available in the database submission.*
Genome browser session<br>(e.g. UCSC)	*Provide a link to an anonymized genome browser session for "Initial submission" and "Revised version" documents only, to enable peer review. Write "no longer applicable" for "Final submission" documents.*

Methodology

Replicates	*Describe the experimental replicates, specifying number, type and replicate agreement.*
Sequencing depth	*Describe the sequencing depth for each experiment, providing the total number of reads, uniquely mapped reads, length of reads and whether they were paired- or single-end.*
Antibodies	*Describe the antibodies used for the ChIP-seq experiments; as applicable, provide supplier name, catalog number, clone name, and lot number.*
Peak calling parameters	*Specify the command line program and parameters used for read mapping and peak calling, including the ChIP, control and index files used.*
Data quality	*Describe the methods used to ensure data quality in full detail, including how many peaks are at FDR 5% and above 5-fold enrichment.*
Software	*Describe the software used to collect and analyze the ChIP-seq data. For custom code that has been deposited into a community repository, provide accession details.*

Flow Cytometry

Plots

Confirm that:

☐ The axis labels state the marker and fluorochrome used (e.g. CD4-FITC).

☐ The axis scales are clearly visible. Include numbers along axes only for bottom left plot of group (a 'group' is an analysis of identical markers).

☐ All plots are contour plots with outliers or pseudocolor plots.

☐ A numerical value for number of cells or percentage (with statistics) is provided.

Methodology

Sample preparation	*Describe the sample preparation, detailing the biological source of the cells and any tissue processing steps used.*
Instrument	*Identify the instrument used for data collection, specifying make and model number.*
Software	*Describe the software used to collect and analyze the flow cytometry data. For custom code that has been deposited into a community repository, provide accession details.*
Cell population abundance	*Describe the abundance of the relevant cell populations within post-sort fractions, providing details on the purity of the samples and how it was determined.*
Gating strategy	*Describe the gating strategy used for all relevant experiments, specifying the preliminary FSC/SSC gates of the starting cell population, indicating where boundaries between "positive" and "negative" staining cell populations are defined.*

☐ Tick this box to confirm that a figure exemplifying the gating strategy is provided in the Supplementary Information.

Magnetic resonance imaging

Experimental design

Design type	Structural MRI (T1-weighted scans)
Design specifications	NA
Behavioral performance measures	NA

Acquisition

Imaging type(s)	https://www.humanconnectome.org/hcp-protocols-ya-3t-imaging, https://github.com/mezera/mrQ, https://community.ukbiobank.ac.uk/hc/en-gb/articles/24618819821981-Imaging-Data
Field strength	3T
Sequence & imaging parameters	See links above for parameters
Area of acquisition	Head

Diffusion MRI ☐ Used ☒ Not used

Preprocessing

Preprocessing software	See links above in acquisition for pipeline information
Normalization	*If data were normalized/standardized, describe the approach(es): specify linear or non-linear and define image types used for transformation OR indicate that data were not normalized and explain rationale for lack of normalization.*
Normalization template	*Describe the template used for normalization/transformation, specifying subject space or group standardized space (e.g. original Talairach, MNI305, ICBM152) OR indicate that the data were not normalized.*
Noise and artifact removal	*Describe your procedure(s) for artifact and structured noise removal, specifying motion parameters, tissue signals and physiological signals (heart rate, respiration).*
Volume censoring	*Define your software and/or method and criteria for volume censoring, and state the extent of such censoring.*

Statistical modeling & inference

Model type and settings	Offline models of volumetry and age, two sided.
Effect(s) tested	Effect of Age on neocortical volume, cerebellar volume, and cognition. Moderation effect of cerebellar volume on age-cognition associations.

Specify type of analysis: ☐ Whole brain ☐ ROI-based ☒ Both

Statistic type for inference (See Eklund et al. 2016)	Both voxel-wise and cluster correction in VBM maps.
Correction	FWE, FDR

Models & analysis

n/a	Involved in the study
☒	☐ Functional and/or effective connectivity
☒	☐ Graph analysis
☒	☐ Multivariate modeling or predictive analysis

Functional and/or effective connectivity	*Report the measures of dependence used and the model details (e.g. Pearson correlation, partial correlation, mutual information).*
Graph analysis	*Report the dependent variable and connectivity measure, specifying weighted graph or binarized graph, subject- or group-level, and the global and/or node summaries used (e.g. clustering coefficient, efficiency, etc.).*
Multivariate modeling and predictive analysis	*Specify independent variables, features extraction and dimension reduction, model, training and evaluation metrics.*
